# Adhesive polypeptides of *Staphylococcus aureus *identified using a novel secretion library technique in *Escherichia coli*

**DOI:** 10.1186/1471-2180-11-117

**Published:** 2011-05-27

**Authors:** Riikka Kylväjä, Matti Kankainen, Liisa Holm, Benita Westerlund-Wikström

**Affiliations:** 1 Division of General Microbiology, Department of Biosciences, University of Helsinki, P.O. Box 56 (Viikinkaari 9C), FIN-00014 University of Helsinki, Finland; 2 Institute of Biotechnology, University of Helsinki, P.O. Box 56 (Viikinkaari 5), FIN-00014, Helsinki, Finland; 3 VTT Technical Research Centre of Finland, P.O. Box 1000, FIN-02044 VTT, Espoo, Finland; 4 Division of Genetics, Department of Biosciences, University of Helsinki, P.O. Box 56 (Viikinkaari 5), FIN-00014, Helsinki, Finland

## Abstract

**Background:**

Bacterial adhesive proteins, called adhesins, are frequently the decisive factor in initiation of a bacterial infection. Characterization of such molecules is crucial for the understanding of bacterial pathogenesis, design of vaccines and development of antibacterial drugs. Because adhesins are frequently difficult to express, their characterization has often been hampered. Alternative expression methods developed for the analysis of adhesins, e.g. surface display techniques, suffer from various drawbacks and reports on high-level extracellular secretion of heterologous proteins in Gram-negative bacteria are scarce. These expression techniques are currently a field of active research. The purpose of the current study was to construct a convenient, new technique for identification of unknown bacterial adhesive polypeptides directly from the growth medium of the *Escherichia coli *host and to identify novel proteinaceous adhesins of the model organism *Staphylococcus aureus*.

**Results:**

Randomly fragmented chromosomal DNA of *S. aureus *was cloned into a unique restriction site of our expression vector, which facilitates secretion of foreign FLAG-tagged polypeptides into the growth medium of *E. coli *Δ*fliC*Δ*fliD*, to generate a library of 1663 clones expressing FLAG-tagged polypeptides. Sequence and bioinformatics analyses showed that in our example, the library covered approximately 32% of the *S. aureus *proteome. Polypeptides from the growth medium of the library clones were screened for binding to a selection of *S. aureus *target molecules and adhesive fragments of known staphylococcal adhesins (e.g coagulase and fibronectin-binding protein A) as well as polypeptides of novel function (e.g. a universal stress protein and phosphoribosylamino-imidazole carboxylase ATPase subunit) were detected. The results were further validated using purified His-tagged recombinant proteins of the corresponding fragments in enzyme-linked immunoassay and surface plasmon resonance analysis.

**Conclusions:**

A new technique for identification of unknown bacterial adhesive polypeptides was constructed. Application of the method on *S. aureus *allowed us to identify three known adhesins and in addition, five new polypeptides binding to human plasma and extracellular matrix proteins. The method, here used on *S. aureus*, is convenient due to the use of soluble proteins from the growth medium and can in principle be applied to any bacterial species of interest.

## Background

Bacterial adhesive proteins, proteinaceous adhesins, are frequently the most critical factor at the onset of a bacterial infection [1-3]. The identification and characterization of such adhesins at the molecular level is therefore crucial for the detailed understanding of bacterial pathogenesis, for the design of vaccines and for the development of novel antibacterial drugs [4,5]. Although some bacterial adhesins have successfully been produced on a large scale and described in detail (for examples the reader is referred to recent reviews and original publications [1-3]), this type of molecules are often difficult to express by conventional techniques or they possess a complicated structure [6]. This has in many cases hampered further characterization of bacterial adhesins and various surface display techniques and alternative expression methods have been developed for the analysis of adhesive polypeptides. However, commonly used surface display techniques suffer from the drawback that they rely on the attachment of the gene product of interest to the surface of the carrier, for example the phage [7], the bacterium [8,9], or the ribosome [10], which may compromise correct folding of the polypeptide of interest. Reports on high-level extracellular secretion of heterologous proteins in Gram-negative bacteria are scarce and these expression techniques are currently a field of active research [11,12].

The adhesion of the important and highly versatile human pathogenic bacterium *Staphylococcus aureus *to host surfaces is mediated by a range of adhesins, some of which are very well characterized [13]. The majority of *S. aureus *adhesins belong to the group of microbial surface components recognizing adhesive matrix molecules, MSCRAMMs [3,14], whereas others represent secretable expanded repertoire adhesive molecules [15]. Some of the known *S. aureus *adhesins have been identified by phage display based on staphylococcal genomic libraries, a technique also used for identification of secreted proteins of the bacterium [16-19]. Bacterial surface display and ribosome display have been exploited for the mapping of *S. aureus *epitopes recognized by human antibodies and for the identification of peptide motifs that mediate entry into eukaryotic cells [20-22]. Nevertheless, on the basis of genomics and proteomics data, a number of surface proteins and approximately 1000 proteins of unknown function in the proteome of *S. aureus *remain to be characterized [13,23] and among these also lie putative novel adhesins.

We recently described an efficient technique for the secretion of foreign proteins into the growth medium of a secretion-competent derivative of the *Escherichia coli *K12-strain called MKS12 [24]. The genes encoding the flagellin (FliC), the flagellar cap (FliD), and the common type 1 fimbriae have been deleted from the chromosome of this strain. Secretion is facilitated by the use of an expression-secretion cassette that includes DNA elements from the flagellin operon of *E. coli*.

In the current report, we further develop the secretion technique [24] into a tool for molecular microbiology and biotechnology and demonstrate its application for the human pathogenic bacterium *S. aureus*. We chose the versatile and important pathogen *S. aureus *as a model organism and constructed a library of random FLAG-tagged staphylococcal polypeptides in the secretion-competent host *E. coli *MKS12 (Δ*fliCfliD*). We sequenced all the inserts carrying a FLAG-encoding sequence and screened the FLAG-tagged polypeptides directly from cell-free growth medium for adhesive properties. The majority of the secreted polypeptides did not bind to the tested target molecules, but we identified totally eight adhesive polypeptides from the library. As a result, we were able to generate a technique, which allows rapid screening of novel bacterial polypeptides directly from the growth medium of *E. coli*.

## Results

### Construction of a primary genomic library of *S. aureus *in *E. coli*

**Figure 1:**
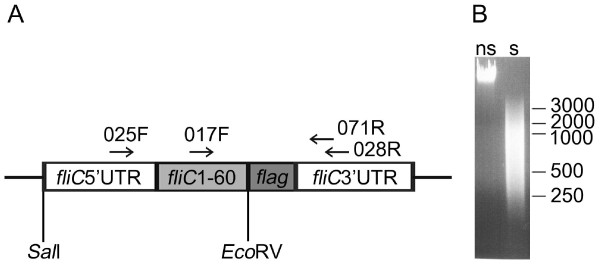
**Elements used in construction of the polypeptide secretion library of *S. aureus* in *E. coli***. A. Expression vector pSRP18/0 contains an expression cassette comprised of a 5' untranslated sequence upstream of the flagellin gene of *E. coli *MG1655 (*fliC*_MG1655_) here indicated *fliC*5'UTR, a DNA fragment encoding the N-terminal 20 amino acids *fliC*_MG1655 _(*fliC*1-60), a synthetic FLAG tag encoding sequence (*flag*) and a 3' untranslated region downstream of *fliC*_MG1655 _(*fliC*3'UTR). *Eco*RV indicates the unique cloning site for foreign DNA fragments, horizontal arrows indicate the oligonucleotides used as primers for PCR (017F, 025F and 028R) and sequencing (017F and 071R) of the cloned inserts and black lines indicate sequences of the plasmid pBR322. *Sal*I and *Bam*HI indicate the restriction sites created during cloning of the expression cassette into pBR322. B. Agarose gel electrophoretic analysis of the chromosomal DNA isolated from *S. aureus *NCTC 8325-4 and used in generation of the library. The purified DNA is shown in the left lane, randomly fragmented and blunted DNA in the right lane. Ns indicate not sonicated and s stands for sonicated and polished DNA. The positions of molecular weight markers in base pairs are shown to the right.

We constructed the vector pSRP18/0 (Figure 1A) carrying the expression-secretion cassette previously shown to efficiently facilitate secretion of heterologous polypeptides in *E. coli *MKS12 [24]. An *Eco*RV restriction site was inserted for cloning of blunt-ended DNA fragments between the DNA fragment carrying nucleotides 1-60 of the *fliC *gene (*fliC*1-60), which in our previous work has been shown to facilitate extracellular secretion of heterologous proteins in *E. coli *MKS12 [24], and the FLAG-tag encoding sequence [25] added for later screening purposes; a stop codon was added at the 3' end of the *flag *sequence.

Purified chromosomal DNA from *S. aureus *subsp. *aureus *(from now on called *S. aureus*) strain NCTC 8325-4 [26] was sonicated into fragments mainly 250 to 1000 bp in length (Figure 1B). The polished, blunt-ended DNA fragments were ligated into pSRP18/0 and transformed into the secretion-competent strain *E. coli *MKS12 to generate a primary genomic library including more than 80 000 colonies. By colony PCR, the cloning efficiency, i.e. the% insert-carrying transformants of all transformants, was estimated from 200 randomly picked colonies to be 60% and the average insert size of 200 randomly picked insert-containing clones was estimated to be approximately 400 bp. The PCR primers used are shown in Figure 1A.

### Generation of the final FLAG-tag positive (Ftp) library in *E. coli*

The 80 000 colonies of the primary genomic library were screened by colony blotting using anti-FLAG antibodies for exclusion of transformants carrying an empty vector or insertions out-of-frame in relation to the FLAG tag. Totally 1663 clones were confirmed to carry gene products with C-terminal FLAG tags and these were included into the final Ftp library. Colony-blot analysis showed that MKS12 (pSRP18/0) with the empty vector reacted with monoclonal anti-FLAG antibodies as weakly as MKS12 carrying no plasmid (data not shown), thus confirming that the Ftp colonies did possess an insertion in their plasmids.

### Sequence analysis of the Ftp library

The coverage of the Ftp library was determined by sequencing the inserted DNA fragments in both directions in all the 1663 Ftp library clones. The sequencing primers are shown in Figure 1A. The sequence of the insert was successfully determined in 1514 clones using the 017F primer and in 1564 clones with the 071R primer. When projected over the genome sequence of *S. aureus *NCTC 8325 using genomic blast searches [27], the 1514 sequences obtained using the 017F primer corresponded to 708963 nt in total and covered 435809 nt of the genome. For the later 1564 sequences obtained with the 071R oligonucleotide, the corresponding values were 769323 nt and 462172 nt, respectively. The sequenced inserts overlapped totally 345890 nts of the genome, thus the overlap of the Ftp library was 63%.

**Figure 2:**
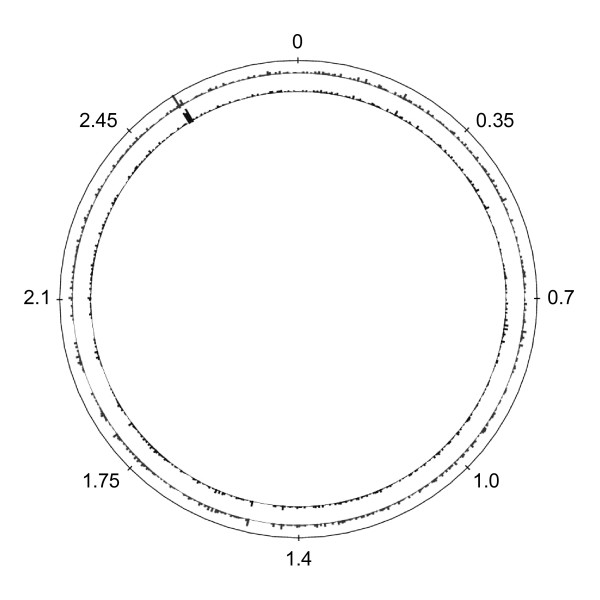
**Distribution of DNA fragments of the Flag-tag positive library clones on the *S. aureus* chromosome**. The height of the bars represents the density of matches in windows of 4 kbp for the first sequence batch obtained with primer 017F (innermost circle) and the second sequence batch obtained using primer 071R (middle circle). The size of the chromosome is 2.82 Mbp (outermost circle); coordinates of the chromosome are indicated in Mbp.

Comparison of the Ftp library sequences with the gene sequences of *S. aureus *NCTC 8325 using BLASTN revealed a significant match for 1325 and 1401 of the 1514 and 1564 determined insertion sequences. The inserts showed homology to 808 and 845 gene sequences, respectively, and covered in total 950 gene sequences in *S. aureus *NCTC 8325. The matches were distributed randomly and evenly over the staphylococcal chromosome (Figure 2). Based on genomic and proteomic data, the theoretical number of encoded proteins in *S. aureus *NCTC 8325 is 2891 [28,29], which indicates that our final Ftp library covers approximately 32% of the staphylococcal proteome. In comparison to advanced but laborious proteomic methods [30] this coverage can be considered reasonable and most of all, it could have been increased by construction and screening of a larger primary genomic library, which had created a higher number of Ftp clones. For a summary of the sequence data obtained from the Ftp library, see Additional file 1 Table S1, which shows that several gene fragments encoding polypeptides of known staphylococcal adhesins such as IgG-binding proteins Protein A and Sbi, fibronectin-binding protein A (FnBPA), clumping factors A and B, elastin-binding protein EbpS, extracellular matrix (ECM) -binding proteins Ebh and Emp, the SD-rich fibrinogen-binding protein as well as enolase [3,13,31] were present in the library.

Nucleotide sequencing of the Ftp clones also showed that three types of inserts existed (examples are presented in Table 1). In the optimal cases, which represented 31% of the Ftp library, the clones carried only one staphylococcal gene or gene fragment which was in the same reading frame as the FliC fragment, added to the construct to facilitate extracellular secretion, and the FLAG-tag. This type of constructs was exemplified by clones named ΔNarG, ΔFnBPA, ΔEbh and ΔCoa. In another case, the staphylococcal gene was in the same reading frame only with the FLAG-tag rendering a gene product without an N-terminal FliC sequence. In the third type of clones several staphylococcal ORFs were identified in the cloned DNA fragment; e.g. two in the clones named ΔPurK, ΔSCOR, ΔUsp and ΔIspD or three in the clone named ΔPBP, although only the distal gene product carried the FLAG tag. We hypothesize that the translation of a FLAG-tag positive gene product in the later two cases, which represented 69% of the library clones, proceeds from the staphylococcal ribosomal binding site (RBS) detected in the 5' untranslated region (5'UTR) of the ORF closest to the FLAG-tag encoding sequence. Hence, the expressed product would be encoded by the last gene fragment of the cloned DNA sequence, would not carry the N-terminal FliC sequence, but would be FLAG-tag positive. Phage display results obtained by Rosander and coworkers [18] as well as our results from sequencing and Western blot analysis (Figure 3A) of selected library clones support the hypothesis of translation of the FLAG-positive gene products from a staphylococcal RBS in *E. coli*. The FLAG-tagged polypeptides observed in the cells of clones ΔPBP, ΔPurK, ΔSCOR, ΔUsp and ΔIspD did not react with anti-flagella antibodies whereas the polypeptides of clones ΔNarG, ΔFnBPA, ΔCoa and ΔEbh did react (data not shown). This result further supports the hypothesis of translation starting from staphylococcal RBSs.

**Table 1:** Examples of Ftp library clones that express adhesive polypeptides

Clone Name	Length of insert*	Chromosomal location of insert^†^	ORFs^‡ ^in insert	Predicted gene product(s) of the Ftp-clone	Presence of FliC_1-20 _and/or FLAG-tag in the gene product	Binding specificity of the product	Predicted molecular mass^#^
ΔNarG	393	2465481-2465873	**1) 02681**	**NarG**^§1^	**FliC_1-20_** **FLAG-tag**	**None**	**18.5**
ΔFnBPA	346	2581863-2582208	**1) 02803**	**FnBPA**^§2^	**FliC_1-20_** **FLAG-tag**	**Fn**	**16.6**
ΔEbh	582	1398633-1399214	**1) 01447**	**Ebh**^§2^	**FliC_1-20_** **FLAG-tag**	**Fn**	**24.2**
ΔCoa	825	212434-213258	**1) 00192**	**coagulase**	**FliC_1-20_** **FLAG-tag**	**Fg, Fn**	**34.2**
ΔPurK	383	979768-980150	1) 01008	out of frame^¶^	No		
			**2) 01009**	**PurK ^§1^**	**FLAG-tag**	**Fn, Fg**	**14.6**
ΔSCOR	484	2667518-2668001	1) 02897	terminator in sequence	FliC		
			**2) 02898**	**Putative SCOR^ §1^**	**FLAG-tag**	**Fn, Fg**	**17.7**
ΔUsp	664	1724620-1725283	1) 01818	out of frame^¶^	No		
			**2) 01819**	**Usp**^§1 ^**-like**	**FLAG-tag**	**Fn, Fg, CIV**,	**19.3**
ΔIspD	885	244692-245576	1) 00223	out of frame^¶^	No		
			**2) 00225**	**IspD**^§2^	**FLAG-tag**	**Fn, Fg**	**13.4**
ΔPBP	756	2257336-2258091	1) 02433	out of frame^¶^	No		
			2) 02432	out of frame^¶^	No		
			**3) 02430**	**putative PBP**^§1^ **of ABC**^§1^**transporter**	**FLAG-tag**	**Fn, Fg**	**6.7**

* In base pairs

† In *S. aureus subsp. aureus NCTC 8325*

‡ Open reading frames (ORFs) in the clones are partial, the number refers to the systematic gene identifier SAOUHSC_no. in the GenBank database, a locus_tag

§1 Abbreviations of TIGR Family names: NarG, nitrate reductase α-subunit; PurK, Phosphoribosylamino-imidazole carboxylase ATPase subunit; SCOR, short-chain oxidoreductase; Usp, universal stress protein family; PBP, periplasmic binding protein; ABC, ATP-binding cassette

§2 Abbreviations of the protein names: FnBPA, fibronectin binding protein A; Ebh, extracellular matrix binding protein homologue; IspD, 2-C-methyl-D-erythritol 4-phosphate cytidylyltransferase

^¶ ^The reading frame is in relation to *fliC *and *flag *sequences

# Molecular mass in kilodaltons. The molecular mass of FliC_1-20 _and FLAG-tag included when present in the gene product

**Figure 3:**
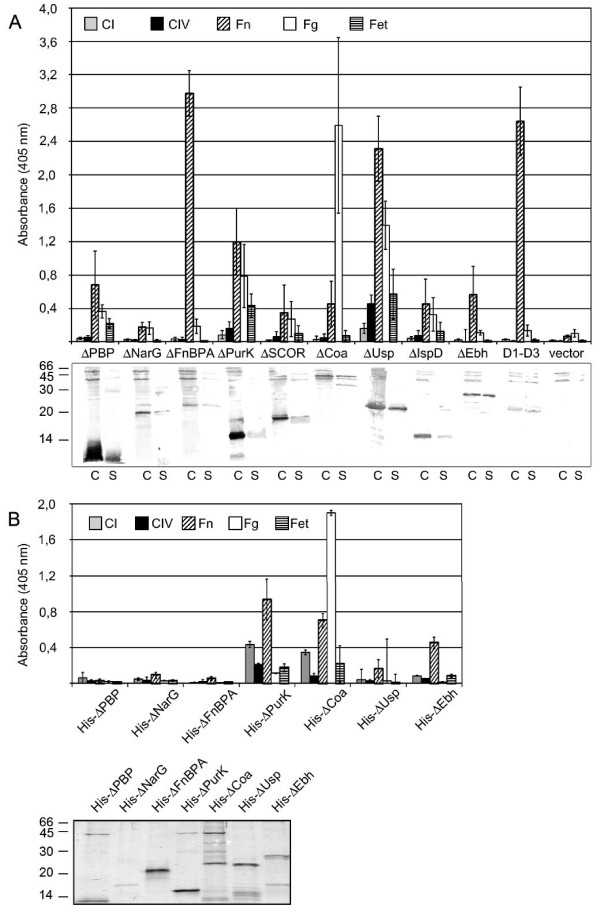
**Properties of polypeptides secreted into the growth medium by the Ftp library clones and purified His-recombinant polypeptides**. A. Upper panel shows the binding of cell-free growth media from the library clones to ECM proteins and the control protein fetuin immobilized in polystyrene microtitre wells as analyzed by ELISA. Lower panel shows Western blot analysis with monoclonal anti-FLAG antibodies of bacterial cells (C) and TCA-precipitated cell-free growth media (S) of the corresponding clones. Vector indicates growth medium from MKS12 (pSRP18/0), D1-D3 denotes polypeptides secreted by MKS12 (pSRP18/0D1-D3), and the names indicate individual library clones. The cell samples correspond to 50 μl and the supernatants to 500 μl bacterial culture, except in the case of clones ΔPBP, ΔUsp, ΔIspD, ΔEbh where supernatant samples corresponding only to 100 μl of culture were loaded due to the high expression level of the polypeptide. B. Upper panel presents the binding of His-tagged recombinant polypeptides to ECM proteins immobilized in polystyrene microtiter wells as analyzed by ELISA and the lower panel shows SDS-PAGE analysis of affinity-purified recombinant polypeptides. The names following His-indicate polypeptides encoded by gene fragments subcloned from corresponding individual library clones. The values are averages of 2 to 3 parallels from 2 to 4 individual experiments, showing the standard deviation as error bars. CI, type I collagen; CIV, type IV collagen; Fn, fibronectin; Fg, fibrinogen; Fet, control protein fetuin. Molecular masses in kDa are indicated to the left.

### Adhesive properties of FLAG-tagged polypeptides in cell-free growth media of Ftp library clones

With the goal to detect known and novel staphylococcal proteinaceous adhesins but on the other hand also to test the applicability of the technique, we analyzed in an enzyme-linked immunoassay (ELISA) the binding of cell-free growth media of the 1663 Ftp library clones to a restricted selection of purified human proteins, which are well-known staphylococcal ligand molecules. These target proteins, i.e. fibrinogen (Fg), plasma fibronectin (Fn), type I and type IV collagens (CI and CIV) as well as the control protein fetuin (Fet), were immobilized in polystyrene microtitre wells and cell-free culture media of the library clones were allowed to bind. Of the totally 1663 clones tested, the polypeptides in the supernatants of eight clones bound to Fn (ΔPBP, ΔFnBPA, ΔPurK, ΔSCOR, ΔCoa, ΔUsp, ΔIspD, ΔEbh) and six to Fg (ΔPBP, ΔPurK, ΔSCOR, ΔCoa, ΔUsp, ΔIspD). The polypeptides in the supernatant of clone ΔUsp interacted with CIV similarly as with the control protein Fet. The binding properties are shown in the upper panel of Figure 3A. The supernatants of the remaining 1655 clones and of the vector strain showed no binding to the tested target proteins, functioned as internal negative controls, and thus indicated specificity in the binding assays. In Figure 3A, clone ΔNarG represents an example of clones expressing non-binding polypeptides; D1-D3 represents polypeptides expressed by MKS12 (pSRP18/0D1-D3) and was included as a Fn-binding positive control [32].

According to our sequence and binding data, three of the Ftp clones expressed adhesive polypeptides previously characterized as adhesins of *S. aureus*, namely the Fn-binding repeats D1-D3 of the Fn-binding protein FnBPA (the clone named ΔFnBPA), a Fn-binding fragment of the ECM-binding protein Ebh (named ΔEbh) and a Fg-binding fragment of staphylocoagulase (named ΔCoa) [32-34]. The coagulase fragment includes the conserved central region and 15 residues of the 27 amino-acids long repeat 1 of coagulase. In group A streptococci, individual repeats of coagulase have been shown to bind Fg and we therefore speculate that the short fragment of repeat 1 mediates the Fg binding we observed [35,36].

The remaining five Ftp clones, which secreted adhesive polypeptides, encoded mainly Fn- or Fg-binding gene products. According to the sequence data, these Ftp-polypeptides were i) an N-terminal fragment of the substrate binding protein of an iron compound ABC transporter (in clone named ΔPBP), ii) an N-terminal fragment of the ATPase subunit of phosphoribosyl aminoimidazole carboxylase (in clone ΔPurK), iii) an N-terminal fragment of a putative short chain oxidoreductase (in clone ΔSCOR), iv) a putative universal stress protein (in clone ΔUsp), and v) the N-terminal half of 2-C-methyl-D-erythritol 4-phosphate cytidylyltransferase (in clone ΔIspD) of *S. aureus *NCTC 8325 [29,37-39]. The gene product of the non-adhesive control clone turned out to be a central fragment of the α-subunit of nitrate reductase and was named ΔNarG [29].

### Western blot analysis of the cell-free growth medium from Ftp clones

To determine the apparent molecular mass of the Ftp polypeptides expressed by the Ftp library clones and to confirm the presence of the C-terminally FLAG-tagged peptides in the growth medium, we analyzed whole cells and cell-free growth media of the clones by Western blotting using anti-FLAG antibodies. The results are presented in the lower panel of Figure 3A and show that the FLAG-tagged gene products were detected in whole cell samples (C) and cell-free supernatants (S), but in varying amounts in each clone. The apparent molecular mass of the secreted polypeptides was in good agreement with their theoretical molecular mass calculated on the basis of the deduced amino acid sequence (Table 1). The FLAG-tagged polypeptide expressed by the clone ΔCoa has however a predicted molecular mass of 34.2 kDa whereas the apparent molecular mass was approximately 45 kDa. The reason for this aberrant migration pattern is unknown, but it is not related to a high content of acidic amino acids causing a slow migration pattern in SDS-PAGE as reported with some other staphylococcal adhesins [40].

### Verification of the adhesive polypeptides

To confirm the results obtained with supernatants of the Ftp library clones, the DNA sequences identified as encoding the adhesive polypeptides (Table 1) were expressed in the cytoplasm of *E. coli *as recombinant polypeptides with six histidine residues at their N-termini by conventional methods. The purified polypeptides (His-ΔPBP, His-ΔNarG, His-ΔFnBPA, His-ΔPurK, His-ΔCoa, His-ΔUsp and His-ΔEbh) are shown in the lower panel of Figure 3B. The concentration of the His-polypeptides was first determined from Coomassie-stained SDS-PAGE gels by analysis of whole band intensity of the corresponding polypeptide using image analysis with an internal protein standard of known concentration. The polypeptides were then assessed for binding to immobilized target molecules by ELISA (at a concentration of 20 nM) and surface plasmon resonance (SPR) analysis (at 0.5-2.5 μM concentrations). His-ΔNarG and His-ΔFnBPA polypeptides were used as internal negative and positive controls, respectively. Since the His-ΔSCOR and His-ΔIspD polypeptides remained insoluble in the *E. coli *cytoplasm, these proteins could not be purified in non-denaturing conditions and could unfortunately not be included in the verification.

In the ELISA assay, the His-ΔCoa and His-ΔEbh polypeptides interacted with the same immobilized target molecules (upper panel of Figure 3B) as those of the corresponding Ftp library clones (upper panel of Figure 3A). The His-ΔPurK polypeptide bound to Fn but interacted poorly with Fg, whereas His-ΔUsp showed only a low level interaction with Fn. Similarly as the negative control polypeptide His-ΔNarG, the His-ΔFnBPA and His-ΔPBP polypeptides showed no binding to Fn or Fg in the ELISA.

In the SPR analysis, the His-ΔPurK, His-ΔCoa, and His-ΔUsp polypeptides bound to immobilized Fg whereas the His-ΔFnBPA, His-ΔPurK, and His-ΔEbh polypeptides showed affinity to Fn similarly as did the cell free growth media of corresponding Ftp library clones tested by ELISA (Figure 3A). In contrast to the ELISA results, the His-ΔEbh polypeptide reacted also with Fg in the SPR analysis. The His-ΔPBP polypeptide and the negative control peptide His-ΔNarG showed no binding properties in the SPR analysis. However, the SPR results mainly confirmed the results obtained with culture supernatants of Ftp clones. The affinity constants obtained in the SPR analysis are shown in Table 2.

**Table 2:** SPR analysis of His_6_-polypeptides

Polypeptide	KD to Fn (M) *	KD to Fg (M) *
His-ΔNarG	0,77	0,72
His-ΔFnBPA	**5,24 × 10^-6^**	0,31
His-ΔEbh	0,02	**1,25 × 10^-6^**
His-ΔCoa	< 0^†^	**1,80 × 10^-7^**
His-ΔPurK	**4,43 × 10^-7^**	**5,39 × 10^-6^**
His-ΔUsp	0,35	**6,45 × 10^-6^**
His-ΔPBP	0,36	0,13

* the steady state affinity constants (KD) of the seven analytes tested are shown in molar concentrations; values shown in bold indicate high affinity for the indicated ligand (Fn or Fg).

† affinity was not measurable since all values were negative

## Discussion

*S. aureus *NCTC 8325, the parental strain of the prophage-cured *S. aureus *NCTC 8325-4 used for construction of the extracelluar secretion library, carries 22 of the genes encoding the 24 surface proteins implicated in adhesion and all the 13 genes for the secretable proteins implicated in immune response evasion as recently described by McCarthy and Lindsay [41]. According to the literature, only eight of these proteins have been reported to bind Fn and/or Fg and five interact with the ECM. Cna, the only collagen-binding protein in the list of adhesins, is not present in *S. aureus *NCTC 8325-4 [41]. Taking into consideration the above data and the fact that we deliberately screened for binding to only a few model targets of *S. aureus*, the yield from our Ftp library was very satisfying. Among the 1663 clones of the Ftp library, which was constructed in the study, we identified in the primary screening for Fn-, Fg- or collagen-binding polypeptides totally eight clones expressing adhesive FLAG-tagged polypeptides. We found three known Fn/Fg-binding polypeptides (ΔFnBPA, ΔEbh and ΔCoa) and in addition five polypeptides of novel adhesive function (ΔPurK, ΔUsp, ΔSCOR, ΔIspD and ΔPBP). The cloned chromosomal fragments frequently encoded polypeptides below the length of intact binding domains of large staphylococcal adhesins, such as the clumping factors (ClfA, ClfB) and SD-rich fibrinogen-binding proteins [14,42]. Hence, in future applications of the presented technique longer chromosomal fragments should preferentially be cloned. We did however identify several fibronectin-binding polypeptides, which possibly is explained by the fact that short fragments of typical fibronectin-binding MSCRAMMs mediate high-affinity binding [43]. The observed variation in concentration of FLAG-tagged polypeptides in the cell-free supernatants of the Ftp-library clones, which was due to variable expression of the cloned *S. aureus *chromosomal fragments in *E. coli *and may have an effect on the screening results, could be circumvented by quantification of the polypeptides prior to the analysis.

The findings obtained by primary screening of Ftp library clones were confirmed by ELISA and SPR analyses using corresponding purified His-tagged recombinant polypeptides. All the binding results are combined in Table 3, and strongly indicate that the Fn- and Fg-binding polypeptides ΔFnBPA, ΔPurK, ΔCoa, ΔUsp and ΔEbh truly have adhesive functions under the tested conditions. The very weak interactions observed with ΔPBP (with Fn and Fg) and ΔUsp (with CIV) require further verifications and could not be confirmed by ELISA or SPR using 6xHis polypeptides. Some discrepancies were observed with the Ebh polypeptides, which may be due to the protein itself or the methods applied for verification of the results. In the ELISA assays, ΔEbh and His-ΔEbh bound to Fn, whereas interaction with Fn as well as Fg was observed in the SPR analysis. Fg is not known to be a ligand for Ebh in the literature, but Ebh is a giant protein, 9535 amino acid residues in length [34], and may have unknown properties. ELISA is an end-point type of analysis, whereas SPR is a real-time analysis considered to be very sensitive and optimal for detection of weak interactions [44]. Thus, the SPR technology may in this case have revealed a novel function of Ebh, which remains to be further characterized in a coming study. The verification of the interactions of ΔSCOR and ΔIspD (with Fn and Fg) was hampered since the polypeptides could not be produced as purified His-tagged polypeptides by conventional expression technology.

**Table 3:** A summary of the binding of *S. aureus *polypeptides to immobilized ligands

Polypeptide tested	ELISA* cell-free supernatant	ELISA* purified 6xHis polypeptide	SPR^† ^purified 6xHis polypeptide
ΔNarG	-	-	-
ΔFnBPA	Fn	-	Fn
ΔEbh	Fn	Fn	(Fn), Fg
ΔCoa	(Fn), Fg	(Fn), Fg,	Fg
ΔPurK	Fn, Fg	Fn, (CI)	Fn, Fg
ΔSCOR	(Fn), (Fg)	ND	ND
ΔUsp	Fn, Fg, (CIV)	(Fn)	Fg
ΔIspD	(Fn), (Fg)	ND	ND
ΔPBP	(Fn), (Fg)	-	-
D1-D3^‡^	Fn	ND	ND

* Binding of polypeptides to immobilized target molecules by enzyme-linked immunoassay

† Binding of polypeptides to immobilized target molecules by surface plasmon resonance

‡ C-terminally FLAG-tagged recombinant polypeptide used as positive control in screening of the FTP library

- indicate no binding; ligand in brackets indicate weak binding

ND, indicate not determined. The SCOR and IspD polypeptides could not be produced as 6xHis recombinant polypeptides and the D1-D3 polypeptide was produced into the cell-free growth medium and did not carry a His tag.

The localization in the *S. aureus *cell of the polypeptides we identified as possessing adhesive properties may appear somewhat controversial. According to bioinformatics analysis and a recent proteomics analysis of the *S. aureus *COL strain [30], the protein PurK, in which we identified an Fg- and Fn-binding polypeptide, is intracellular and functions as the ATPase subunit of phosphoribosylaminoimidazole carboxylase. The Fn-/Fg-binding polypeptides SCOR (a putative short chain oxidoreductase), Usp (a universal stress protein) and IspD (2-C-methyl-D-erythritol 4-phosphate cytidylyltransferase) are found both in the cytoplasm and on the cell surface of *S. aureus *[43]. Finally, the PBP polypeptide (substrate binding protein of an iron compound ABC transporter) has been indicated as a lipoprotein. There is increasing evidence that various bacterial proteins regarded as cytoplasmic enzymes also can be found in other tasks outside the bacterial cell and presumably have a dual role. Several examples of such moonlighting proteins [45] and/or anchorless adhesins [46], for which the secretion mechanism still is unknown, have been reported [47-49]. In addition, screenings for vaccine candidates in *S. aureus *by ribosome display combined with immunoproteome analysis as well as by proteomics-based techniques have identified also intracellular proteins and anchorless cell wall proteins as immunogenic and/or located on the outside of the bacterial cell [22,50-53]. This indicates that some bacterial intracellular proteins may play a role or, alternatively, at least be localized extracellularly during the *in vivo *infection. Hence, it is likely that our results are not *in vitro *artefacts and that the Fn- and Fg-binding Usp and PurK polypeptides we identified, if localized extracellularly, could mediate host-microbe interaction. It should however be stressed, that the adhesive polypeptides were expressed in a heterologous host and for the obtained results to be fully reliable and reflect the native activity of *S. aureus *proteins, the properties demonstrated for these polypeptides should be further verified in a separate study.

A comparison of the presented technique with alternative expression methods applied in analysis of adhesins and/or the immunoproteome of *S. aureus *reveals benefits and deficiencies in all the technologies. Proteomics-based methods rely on proteins expressed by the target organism at the particular condition that may render the expressome incomplete [54], whereas our method in principle facilitates the expression of any gene product independently of the growth requirements of the target bacterium, i.e. *S. aureus *in our case. The application of other commonly used techniques, such as the proteomics-based expression library screening, ribosome display and surface display techniques, suffer from individual drawbacks exemplified by requirement of cell lysis, removal of cell debris prior to analysis, conformation of the polypeptide to be displayed, disulfide bonds disturbing the surface translocation, or the use of expensive commercial *in vitro *transcription and translation kits [8,10,55,56]. A drawback in biotechnological applications of the recently published complete ORFeome library of *S. aureus *is the requirement to transfer the library plasmids into appropriate expression hosts prior to protein production [57].

The most time-consuming part of the method presented here is the manual construction of the final Ftp library. Once the library has been generated, it can conveniently in a cost- and time-efficient manner be applied in the analysis of any protein-ligand interaction directly using cell-free supernatants in various binding assays. A clear advantage of our and other extracellular secretion techniques such as type I and type III secretion-based methods [58-60] is the cheap and convenient direct use of cell-free growth media, whereas techniques dependent on intracellular proteins or proteins exported to the periplasm by the SecA-YEG or Tat pathways are more tedious and expensive [61]. As apparent from our results with the polypeptides His-ΔSCOR and His-ΔIspD, proteins difficult to produce by conventional methods may be efficiently produced by this novel and flexible alternative method.

## Conclusions

In this study, we generated a random chromosomal library of *S. aureus *in the secretion-competent strain *E. coli *MKS12 (Δ*fliCfliD*), selected only the clones that expressed C-terminally Flag-tagged gene products, and sequenced the DNA fragments of all these 1663 clones. The fragments were distributed evenly over the *S. aureus *chromosome and the library covered approximately 32% of the *S. aureus *proteome. We tested the extracellularly secreted staphylococcal polypeptides for binding to well-known ligands of *S. aureus *and found previously characterized adhesins, such as the Fn-binding D1-D3 repeats of FnBPA, a Fg-binding fragment of staphylocoagulase and a Fn-binding fragment of the ECM-binding protein Ebh. Furthermore, we found five polypeptides with new adhesive properties, a polypeptide of the universal stress protein Usp, and adhesive fragments of the putative short chain oxidoreductase SCOR, the phosphoribosylaminoimidazole carboxylase ATPase subunit PurK, 2-C-methyl-D-erythritol 4-phosphate cytidylyltransferase IspD, and the substrate binding protein of an iron compound ABC transporter, which all bound to Fn and Fg. Currently, we are analyzing the library more comprehensively by screening reactivity of Ftp polypeptides immobilized via the FLAG tag with antibodies from healthy individuals and patients suffering from various staphylococcal infections. This methodologically straight-forward method can in principle be applied on any bacterial species and protein-ligand interaction of interest.

## Methods

### Bacterial strains and growth conditions

The host strain *E. coli *MKS12, and *S. aureus *subsp. *aureus *strain NCTC 8325-4 were available from previous work [24,62]. *E. coli *strains were cultured shaking, in Luria broth (LB) or on agar plates supplemented with ampicillin (150 μg/ml) and streptomycin (100 μg/ml) when appropriate, for 18 h at 37°C. For analysis of adhesive properties, the library clones were grown statically on 96-well polystyrene plates in 300 μl LB and for Western blot analysis the bacteria were grown statically in 3 ml LB. *S. aureus *NCTC 8325-4 was grown in tryptic soy broth or on agar for 18 h at 37°C.

### Construction of the library vector

A DNA fragment carrying a 173-bp 5' UTR upstream of the flagellin gene of *E. coli *MG1655 [24], a sequence encoding the 20 N-terminal amino acids (*fliC*_1-60_) of FliC_MG1655_, an *Eco*RV restriction site, a FLAG-tag encoding sequence [25], a stop codon, and a 321-bp 3' UTR of *fliC*_MG1655 _[24] was generated by PCR, digested and ligated into the *Sal*I-*Eco*RV digested plasmid pBR322 [63]. This gave the plasmid pSRP18/0 (Figure 1A), which carries the *flag *sequence in the same reading frame as the *fliC*_1-60_. Chromosomal DNA of *E. coli *MG1655 Δ*fimA-H *[64] used as a template was available from previous work [24] and primers were designed on the basis of the nucleotide sequence of *E. coli *MG1655. The flag sequence (gactacaaggacgatgacgataag), the stop codon TAA, and the restriction sites used in cloning were included in the oligonucleotides used as primers in PCR. Standard recombinant DNA techniques were used [65].

### Construction of the primary genomic library

Chromosomal DNA from *S. aureus *NCTC 8325-4 was purified using Blood and cell culture DNA Midi Kit with genomic-tip 100/G (Qiagen) and randomly fragmented by ultrasonic treatment (4 sec., Ultrasonic processor, VCX600) into fragments of mainly 250 to 1000 bp in length. The DNA fragments were blunted with Mung bean nuclease, the *Eco*RV linearized pSRP18/0 was dephosphorylated with Calf intestinal alkaline phosphatase and the genomic fragments were ligated into pSRP18/0 with T4 DNA ligase using enzymes obtained from Promega according to manufacturer's instructions. The ligation mixture was electroporated into *E. coli *MKS12 and transformants grown on Luria agar plates complemented with antibiotics. This generated the primary genomic library of *S. aureus *NCTC 8325-4 in *E. coli*.

### Generation of the final Ftp peptide library

We screened the 80000 transformants of the primary genomic library by colony blotting using anti-FLAG antibodies and selected for the library only the Ftp clones. Briefly, a 0.45 μm nitrocellulose membrane (Whatman) was placed on top of bacterial colonies grown on Luria plates for 5 minutes. After removal, the membranes were washed once with PBS containing 0.05% Tween™ 20 (v/v), twice with PBS and blocked at 20°C for 1 h in 2% BSA/PBS (w/v), rinsed again in PBS and incubated with antibodies. Anti-FLAG^® ^M2 mAb (Sigma-Aldrich) was diluted in 1% BSA/PBS to a concentration of 0.5 μg/ml and alkaline phosphatase-conjugated secondary antibodies (Dako) to a concentration of 1.5 μg/ml in the same buffer. Ftp clones were picked from the original plates, grown on fresh Luria plates and screened again using the same procedure. On the second round, strain MKS12 (pSRP18/0) was included as a background control and MKS12 expressing D repeats D1-D3 from FnBPA [32] cloned into pSRP18/0 was included as a positive control on the plates. The gene fragment encoding the D1-D3 repeats of the FnBPA protein from *S. aureus *was cloned by PCR into the *Eco*RV site of pSRP18/0 to generate the plasmid p18/0D1-D3. The plasmid pFR015, carrying the *fnbA *gene, was available from previous work [62] and used as a template, the oligonucleotides used as primers were designed on the basis of *fnbA *sequence [32].

### Construction and purification of His-tagged *S. aureus *polypeptides

The gene fragments of the library clones, which encoded an Ftp gene product, were recloned into the pQE30 vector by PCR. Primers were designed on the basis of the sequence obtained from the plasmids of corresponding Ftp clones, which also were used as templates in the PCR. For cloning purposes, the forward primers carried a *Bam*HI or a *Hind*III restriction site and the reverse primers included a *Sph*I or a *Sal*I restriction site. Expression of the gene fragments and purification of the N-terminal His_6_-tagged polypeptides was performed under native conditions according to the QIA *express *System (Qiagen). The purified polypeptides were dialysed against PBS before use and concentration of the correct His-polypeptides was determined from Coomassie-stained SDS-PAGE gels by analysis of whole band intensity of the corresponding polypeptide using image analysis with an internal protein standard of known concentration and using the TINA 2.09c software (Rayest Isotopen Meβgeräte).

### Clarification and precipitation of growth media

The growth medium of library clones cultured in 300 μl LB in 96-well polypropylene plates was centrifuged twice for 15 minutes at 2000 × g and 100 μl of the final supernatant from each well was used for binding assays. For Western blot analysis 1 ml growth medium from a 3 ml bacterial culture was clarified by centrifugation and precipitated with TCA as described before [24].

### Binding assay and Western blotting

Purified human CI, CIV (Becton Dickinson Labware) and plasma Fn (US Biological) were immobilized onto 96-well polystyrene microtiter plates at a final coating concentration of 2 pmol per well in PBS, as described before [66]. Purified Fg (Kordia Life Sciences) and Fet (Sigma-Aldrich) were immobilized at a concentration of 0.85 μg per well for 20 h at 20°C and the wells were subsequently blocked with 2% BSA/PBS for 2 h at 20°C. 100 μl clarified supernatants or 20 nM of purified His-polypeptides were added and left to react with the immobilized proteins for 2 h at +37°C. Bound, extracellularly secreted polypeptides were detected with anti-FLAG^® ^M2 mAb (0.5 μg/ml in 1% BSA/PBS) and bound, purified 6xHis polypeptides with anti-His mAb (0.1 μg/ml in 1% BSA/PBS, Clontech Laboratories). Alkaline phosphatase-conjugated antibodies (1 μg/ml in 1% BSA/PBS, Dako) were used as secondary antibodies, *P*-nitrophenyl phosphate (Sigma-Aldrich) was used as a substrate, and the absorbance was measured in a Multiscan Titertek recorder (Eflab) at 405 nm. Reaction volumes were in all steps 100 μl per well. In Western blotting, samples corresponding to 100 or 500 μl of growth medium and 50 μl bacterial culture were analyzed in a 20% SDS-PAGE gel and transferred onto 0.2 μm nitrocellulose membranes. The detection was done using anti-FLAG antibody (0.5 μg/ml in 1% BSA/PBS) and alkaline phosphatase-conjugated anti-mouse antibody (1.5 μg/ml in 1% BSA/PBS).

### SPR assay

The interaction between purified His-polypeptides and Fn as well as Fg was analyzed by SPR technology using the Biacore T100 instrument, CM5 sensor chips and amine coupling chemistry according to the manufacturer's instructions (GE Healthcare). Single cycle kinetics was applied in the measurements [67]. Briefly, ligands were diluted in sodium acetate, pH 4.5 to 30 μg/ml (Fn) and 80 μg/ml (Fg) and applied onto activated sensor chip surface at flow rates 10 μl/min for 7 min with Fg and 5 μl/min for 9 min with Fn. His-polypeptides used as analytes at concentrations of 0.5 μM, 1.0 μM, 1.5 μM, 2.0 μM and 2.5 μM in PBS were injected at a flow rate of 30 μl/min using PBS as a running buffer. Regeneration of the surface was done between the different analytes using 10 mM glycine, pH 2.3 for Fg and 5 mM NaOH for Fn; control samples were used to confirm that regeneration did not affect the binding.

### PCR screening and sequencing of the clones

Colony PCR was used to estimate the cloning efficiency, i.e. the% insert-carrying transformants of all transformants in the primary genomic library, from 200 randomly picked colonies and to estimate the average insert size of 200 randomly picked insert-containing clones. The colony PCR was performed using Dynazyme II DNA polymerase (Finnzymes), the PCR primers 017F (5' taccaacagcctctcgctg 3') and 028R (5' caattcaacttgtaggcctgata 3') purchased from Medprobe shown in Figure 1A, recombinant bacterial cells as templates, and applying standard recombinant DNA techniques [65]. The insertions in the 1663 Ftp clones were amplified by PCR using the primers 025F (5' ggcgattgagccgacgg 3') and 028R and the recombinant plasmids as templates. The inserts were then sequenced in both directions using the primer 017F for the first sequence batch and primer 071R (5' ataagcgcagcgcatcagg 3') for the second batch (Institute of Biotechnology, University of Helsinki, Finland). The primers, which were designed to flank the cloning site in vector pSRP18/0 using the sequences of *E. coli *MG1655 [68] and pBR322 [69], were purchased from Medprobe or Biomers.

### Bioinformatics analysis of the cloned *S. aureus *sequences

The sequences obtained from the insertions of the Ftp library were compared against the genome and gene sequences of *S. aureus *NCTC 8325 using basic local alignment search tool, BLASTN [27]. By accepting pairwise alignments with at least 95% sequence identity and of length at least 30 nt, a hit was recorded for 1446 and 1538 query sequences in the first and second sequence batch, respectively. All these sequences matched a single genomic region on the genome sequence. In the gene search, query sequences were required to share at least 95% identity and at least 95 nt continuous alignment against the subject sequence. This search resulted in hits for 1325 and 1401 query sequences that showed a trustworthy match against 1695 and 1747 subject sequences. To have a one-to-one correspondence between queries and subjects, we only accepted the gene closest to the *flag *sequence end of the query sequence.

Prediction of amino acid composition and molecular mass on the basis on deduced protein sequences of the library clones was done using ProtParam-tool [70] and analyses of signal sequences were carried out using SignalP and LipoP [71,72]. Gene sequences were also re-annotated by converting them into amino acid sequences, performing a homology search using BLASTP [27] and choosing the most representative descriptions for them with Blannotator [73].

### Accession numbers

*E. coli *MG1655, GenBank: U00096 and NCBI: NC_000913; pBR322, GenBank: J01749; *S. aureus *subsp. *aureus *NCTC 8325, GenBank: CP000253 and NCBI: NC_007795; *fnbA*, GenBank: J04151.

## List of abbreviations

5'UTR: 5' untranslated region; BLAST: basic local alignment search tool; CI: type I collagen; CIV: type IV collagen; ECM: extracellular matrix; ELISA: enzyme-linked immunoassay; Fet: fetuin; Fg: fibrinogen; *fliC*_1-60_: base pairs 1 to 60 of *fliC*; FliC_1-20_: N-terminal 20 amino acids of flagellin; Fn: human plasma fibronectin; Ftp: Flag-tag positive; MSCRAMMs: microbial surface components recognizing adhesive matrix molecules; ORF: open reading frame; PBP: periplasmic binding protein; PELS: proteomics-based expression library screening; RBS: ribosomal binding site; *S. aureus*, *S. aureus *subsp. *aureus*; SPR: surface plasmon resonance.

## Authors' contributions

RK carried out construction of the primary genomic library as well as the Ftp library, screening of the Ftp library for adhesive polypeptides, cloning and expression of His-tagged polypeptides, binding analyses by ELISA and SPR, as well as the SDS-PAGE and Western blot analyses. She also constructed the plasmids, participated in the study design and interpretation of data, and in drafting of the manuscript. MK and LH carried out the bioinformatics analysis of DNA sequence data, participated in the study design and in revising the manuscript critically. BWW coordinated the DNA sequencing, had the main responsibility for the study design, data interpretation and manuscript writing. All authors read and approved the final manuscript.

## Supplementary Material

Additional file 1 **"Table S1" shows the list of gene products found by DNA sequencing and bioinformatics of the Ftp-library**. Examples of known adhesive surface proteins and adhesive polypeptides described in the current report are shown in boldface. The abbreviations used as clone and polypeptide names in the current report are shown in parenthesis. Click here for file

## References

[B1] Proft T Baker EN Pili in Gram-negative and Gram-positive bacteria - structure, assembly and their role in disease Cell Mol Life Sci 2009 66 4 613 635 10.1007/s00018-008-8477-4 18953686 PMC11131518

[B2] Rivera J Vannakambadi G Höök M Speziale P Fibrinogen-binding proteins of Gram-positive bacteria Thromb Haemost 2007 98 3 503 511 17849038

[B3] Speziale P Pietrocola G Rindi S Provenzano M Provenza G Di Poto A Visai L Arciola CR Structural and functional role of *Staphylococcus aureus *surface components recognizing adhesive matrix molecules of the host Future Microbiol 2009 4 1337 1352 10.2217/fmb.09.102 19995192

[B4] Cegelski L Marshall GR Eldridge GR Hultgren SJ The biology and future prospects of antivirulence therapies Nat Rev Microbiol 2008 6 1 17 27 10.1038/nrmicro1818 18079741 PMC2211378

[B5] Rasko DA Sperandio V Anti-virulence strategies to combat bacteria-mediated disease Nat Rev Drug Discov 2010 9 2 117 128 10.1038/nrd3013 20081869

[B6] Niemann HH Schubert WD Heinz DW Adhesins and invasins of pathogenic bacteria: a structural view Microbes Infect 2004 6 1 101 112 10.1016/j.micinf.2003.11.001 14738899

[B7] Paschke M Phage display systems and their applications Appl Microbiol Biotechnol 2006 70 1 2 11 10.1007/s00253-005-0270-9 16365766

[B8] Samuelson P Gunneriusson E Nygren P Ståhl S Display of proteins on bacteria J Biotechnol 2002 96 2 129 154 10.1016/S0168-1656(02)00043-3 12039531

[B9] Majander K Anton L Kylväjä R Westerlund-Wikström B Jarrell KF The bacterial flagellum as a surface display and expression tool Pili and flagella: Current research and future trends 2009 Norfolk UK: Caister Academic Press 191 206

[B10] Yan X Xu Z Ribosome-display technology: applications for directed evolution of functional proteins Drug Discov Today 2006 11 19-20 911 916 10.1016/j.drudis.2006.08.012 16997141

[B11] Choi JH Lee SY Secretory and extracellular production of recombinant proteins using *Escherichia coli* Appl Microbiol Biotechnol 2004 64 5 625 635 10.1007/s00253-004-1559-9 14966662

[B12] Ni Y Chen R Extracellular recombinant protein production from *Escherichia coli* Biotechnol Lett 2009 31 11 1661 1670 10.1007/s10529-009-0077-3 19597765

[B13] Clarke SR Foster SJ Surface adhesins of *Staphylococcus aureus* Adv Microb Physiol 2006 51 187 224 17010697 10.1016/S0065-2911(06)51004-5

[B14] Foster TJ Höök M Surface protein adhesins of *Staphylococcus aureus* Trends Microbiol 1998 6 12 484 488 10.1016/S0966-842X(98)01400-0 10036727

[B15] Chavakis T Wiechmann K Preissner KT Herrmann M *Staphylococcus aureus *interactions with the endothelium: the role of bacterial "secretable expanded repertoire adhesive molecules" (SERAM) in disturbing host defense systems Thromb Haemost 2005 94 2 278 285 16113816 10.1160/TH05-05-0306

[B16] Barbu EM Ganesh VK Gurusiddappa S Mackenzie RC Foster TJ Sudhof TC Höök M beta-Neurexin is a ligand for the *Staphylococcus aureus *MSCRAMM SdrC PLoS Pathog 2010 6 1 e1000726. 20090838 10.1371/journal.ppat.1000726 PMC2800189

[B17] Clarke SR Brummell KJ Horsburgh MJ McDowell PW Mohamad SA Stapleton MR Acevedo J Read RC Day NP Peacock SJ Mond JJ Kokai-Kun JF Foster SJ Identification of in vivo-expressed antigens of *Staphylococcus aureus *and their use in vaccinations for protection against nasal carriage J Infect Dis 2006 193 8 1098 1108 10.1086/501471 16544250

[B18] Rosander A Bjerketorp J Frykberg L Jacobsson K Phage display as a novel screening method to identify extracellular proteins J Microbiol Methods 2002 51 1 43 55 10.1016/S0167-7012(02)00052-0 12069889

[B19] Bjerketorp J Nilsson M Ljungh Å Flock JI Jacobsson K Frykberg L A novel von Willebrand factor binding protein expressed by *Staphylococcus aureus* Microbiology 2002 148 Pt 7 2037 2044 12101292 10.1099/00221287-148-7-2037

[B20] Etz H Minh DB Henics T Dryla A Winkler B Triska C Boyd AP Söllner J Schmidt W von Ahsen U Buschle M Gill SR Kolonay J Khalak H Fraser CM von Gabain A Nagy E Meinke A Identification of in vivo expressed vaccine candidate antigens from *Staphylococcus aureus* Proc Natl Acad Sci USA 2002 99 10 6573 6578 10.1073/pnas.092569199 11997460 PMC124444

[B21] Taschner S Meinke A von Gabain A Boyd AP Selection of peptide entry motifs by bacterial surface display Biochem J 2002 367 Pt 2 393 402 12144529 10.1042/BJ20020164 PMC1222908

[B22] Weichhart T Horky M Söllner J Gangl S Henics T Nagy E Meinke A von Gabain A Fraser CM Gill SR Hafner M von Ahsen U Functional selection of vaccine candidate peptides from *Staphylococcus aureus *whole-genome expression libraries in vitro Infect Immun 2003 71 8 4633 4641 10.1128/IAI.71.8.4633-4641.2003 12874343 PMC166000

[B23] Hecker M Becher D Fuchs S Engelmann S A proteomic view of cell physiology and virulence of *Staphylococcus aureus* Int J Med Microbiol 2010 300 2-3 76 87 10.1016/j.ijmm.2009.10.006 20005169

[B24] Majander K Anton L Antikainen J Lång H Brummer M Korhonen TK Westerlund-Wikström B Extracellular secretion of polypeptides using a modified *Escherichia coli *flagellar secretion apparatus Nat Biotechnol 2005 23 4 475 481 10.1038/nbt1077 15806100

[B25] Javed A Zaidi SK Gutierrez SE Lengner CJ Harrington KS Hovhannisyan H Cho BC Pratap J Pockwinse SM Montecino M Wijnen AJ Lian JB Stein JL Stein GS Immunofluorescence analysis using epitope-tagged proteins: in vitro system Methods Mol Biol 2004 285 33 36 15269394 10.1385/1-59259-822-6:033

[B26] Novick R Properties of a cryptic high-frequency transducing phage in *Staphylococcus aureus* Virology 1967 33 1 155 166 10.1016/0042-6822(67)90105-5 4227577

[B27] Altschul SF Madden TL Schäffer AA Zhang J Zhang Z Miller W Lipman DJ Gapped BLAST and PSI-BLAST: a new generation of protein database search programs Nucleic Acids Res 1997 25 17 3389 3402 10.1093/nar/25.17.3389 9254694 PMC146917

[B28] Hecker M Engelmann S Cordwell SJ Proteomics of *Staphylococcus aureus*--current state and future challenges J Chromatogr B 2003 787 1 179 195 10.1016/S1570-0232(02)00907-8 12659740

[B29] Gillaspy AF Worrell V Orvis J Roe BA Dyer DW Iandolo JJ Fischetti V, Novick R, Ferretti J, Portnoy D, Rood J The *Staphylococcus aureus *NCTC8325 Genome Gram positive pathogens 2006 Washington, DC, USA: ASM Press 381 412

[B30] Becher D Hempel K Sievers S Zühlke D Pané-Farré J Otto A Fuchs S Albrecht D Bernhardt J Engelmann S Völker U van Dijl JM Hecker M A proteomic view of an important human pathogen-towards the quantification of the entire *Staphylococcus aureus *proteome PLoS One 2009 4 12 e8176. 19997597 10.1371/journal.pone.0008176 PMC2781549

[B31] Zhang L Jacobsson K Vasi J Lindberg M Frykberg L A second IgG-binding protein in *Staphylococcus aureus* Microbiology 1998 144 Pt 4 985 991 9579072 10.1099/00221287-144-4-985

[B32] Signäs C Raucci G Jönsson K Lindgren P Anantharamaiah GM Höök M Lindberg M Nucleotide sequence of the gene for a fibronectin-binding protein from *Staphylococcus aureus*: use of this peptide sequence in the synthesis of biologically active peptides Proc Natl Acad Sci USA 1989 86 2 699 703 10.1073/pnas.86.2.699 2521391 PMC286541

[B33] McDevitt D Vaudaux P Foster TJ Genetic evidence that bound coagulase of *Staphylococcus aureus *is not clumping factor Infect Immun 1992 60 4 1514 1523 1548075 10.1128/iai.60.4.1514-1523.1992 PMC257025

[B34] Clarke SR Harris LG Richards RG Foster SJ Analysis of Ebh, a 1.1-Megadalton Cell Wall-Associated Fibronectin-Binding Protein of *Staphylococcus aureus* Infect Immun 2002 70 12 6680 6687 10.1128/IAI.70.12.6680-6687.2002 12438342 PMC133066

[B35] Schubert A Zakikhany K Schreiner M Frank R Spellerberg B Eikmanns BJ Reinscheid DJ A fibrinogen receptor from group B *Streptococcus *interacts with fibrinogen by repetitive units with novel ligand binding sites Mol Microbiol 2002 46 2 557 569 10.1046/j.1365-2958.2002.03177.x 12406229

[B36] Watanabe S Ito T Takeuchi F Endo M Okuno E Hiramatsu K Structural Comparison of Ten Serotypes of Staphylocoagulases in *Staphylococcus aureus* J Bacteriol 2005 187 11 3698 3707 10.1128/JB.187.11.3698-3707.2005 15901693 PMC1112059

[B37] Kvint K Nachin L Diez A Nyström T The bacterial universal stress protein: function and regulation Curr Opin Microbiol 2003 6 2 140 145 10.1016/S1369-5274(03)00025-0 12732303

[B38] Zhang Y Morar M Ealick SE Structural biology of the purine biosynthetic pathway Cell Mol Life Sci 2008 65 23 3699 3724 10.1007/s00018-008-8295-8 18712276 PMC2596281

[B39] Higgins CF ABC transporters: physiology, structure and mechanism--an overview Res Microbiol 2001 152 3-4 205 210 10.1016/S0923-2508(01)01193-7 11421269

[B40] Joh HJ House-Pompeo K Patti JM Gurusiddappa S Höök M Fibronectin receptors from gram-positive bacteria: comparison of active sites Biochemistry 1994 33 20 6086 6092 10.1021/bi00186a007 8193122

[B41] McCarthy AJ Lindsay JA Genetic variation in Staphylococcus aureus surface and immune evasion genes is lineage associated: implications for vaccine design and host-pathogen interactions BMC Microbiol 2010 10 173 10.1186/1471-2180-10-173 20550675 PMC2905362

[B42] Ponnuraj K Bowden MG Davis S Gurusiddappa S Moore D Choe D Xu Y Höök M Narayana SV A "dock, lock, and latch" structural model for a staphylococcal adhesin binding to fibrinogen Cell 2003 115 2 217 228 10.1016/S0092-8674(03)00809-2 14567919

[B43] Schwarz-Linek U Werner JM Pickford AR Gurusiddappa S Kim JH Pilka ES Briggs JAG Gough TS Höök M Campbell ID Potts JR Pathogenic bacteria attach to human fibronectin through a tandem ß-zipper Nature 2003 423 6936 177 181 10.1038/nature01589 12736686

[B44] Avramis VI Avramis EV Hunter W Long MC Immunogenicity of native or pegylated E. coli and Erwinia asparaginases assessed by ELISA and surface plasmon resonance (SPR-biacore) assays of IgG antibodies (Ab) in sera from patients with acute lymphoblastic leukemia (ALL) Anticancer Res 2009 29 1 299 302 19331165

[B45] Jeffery CJ Moonlighting proteins: old proteins learning new tricks Trends Genet 2003 19 8 415 417 10.1016/S0168-9525(03)00167-7 12902157

[B46] Chhatwal GS Anchorless adhesins and invasins of Gram-positive bacteria: a new class of virulence factors Trends Microbiol 2002 10 5 205 208 10.1016/S0966-842X(02)02351-X 11973142

[B47] Tunio SA Oldfield NJ Berry A Ala'aldeen DA Wooldridge KG Turner DP The moonlighting protein fructose-1, 6-bisphosphate aldolase of Neisseria meningitidis: surface localization and role in host cell adhesion Mol Microbiol 2010 10.1111/j.1365-2958.2010.07098.x 20199602

[B48] Hurmalainen V Edelman S Antikainen J Baumann M Lähteenmäki K Korhonen TK Extracellular proteins of *Lactobacillus crispatus *enhance activation of human plasminogen Microbiology 2007 153 Pt 4 1112 1122 17379720 10.1099/mic.0.2006/000901-0

[B49] Pancholi V Chhatwal GS Housekeeping enzymes as virulence factors for pathogens Int J Med Microbiol 2003 293 6 391 401 10.1078/1438-4221-00283 14760970

[B50] Vytvytska O Nagy E Blüggel M Meyer HE Kurzbauer R Huber LA Klade CS Identification of vaccine candidate antigens of *Staphylococcus aureus *by serological proteome analysis Proteomics 2002 2 5 580 590 10.1002/1615-9861(200205)2:5<580::AID-PROT580>3.0.CO;2-G 11987132

[B51] Glowalla E Tosetti B Krönke M Krut O Proteomics-based identification of anchorless cell wall proteins as vaccine candidates against *Staphylococcus aureus* Infect Immun 2009 77 7 2719 2729 10.1128/IAI.00617-08 19364833 PMC2708538

[B52] Dreisbach A Hempel K Buist G Hecker M Becher D van Dijl JM Profiling the surfacome of Staphylococcus aureus Proteomics 2010 10 17 3082 3096 10.1002/pmic.201000062 20662103

[B53] Holtfreter S Kolata J Bröker BM Towards the immune proteome of Staphylococcus aureus - The anti-S. aureus antibody response Int J Med Microbiol 2010 300 2-3 176 192 10.1016/j.ijmm.2009.10.002 19889576

[B54] Ziebandt AK Kusch H Degner M Jaglitz S Sibbald MJ Arends JP Chlebowicz MA Albrecht D Pantuček R Doškar J Ziebuhr W Bröker BM Hecker M van Dijl JM Engelmann S Proteomics uncovers extreme heterogeneity in the *Staphylococcus aureus *exoproteome due to genomic plasticity and variant gene regulation Proteomics 2010 10 8 1634 1644 10.1002/pmic.200900313 20186749

[B55] Kudva IT Krastins B Sheng H Griffin RW Sarracino DA Tarr PI Hovde CJ Calderwood SB John M Proteomics-based expression library screening (PELS): A novel method for rapidly defining microbial immunoproteomes Mol Cell Proteomics 2006 5 8 1514 1519 10.1074/mcp.T600013-MCP200 16737953 PMC2754196

[B56] Henics T Winkler B Pfeifer U Gill SR Buschle M von Gabain A Meinke AL Small-fragment genomic libraries for the display of putative epitopes from clinically significant pathogens BioTechniques 2003 35 1 196 209 12866421 10.2144/03351dd03

[B57] Brandner CJ Maier RH Henderson DS Hintner H Bauer JW Önder K The ORFeome of *Staphylococcus aureus *v 1.1 BMC Genomics 2008 9 321 10.1186/1471-2164-9-321 18605992 PMC2474624

[B58] Gentschev I Dietrich G Goebel W The *E. coli *α-hemolysin secretion system and its use in vaccine development Trends Microbiol 2002 10 1 39 45 10.1016/S0966-842X(01)02259-4 11755084

[B59] Nishikawa H Sato E Briones G Chen L Matsuo M Nagata Y Ritter G Jäger E Nomura H Kondo S Tawara I Kato T Shiku H Old LJ Galán JE Gnjatic S In vivo antigen delivery by a *Salmonella typhimurium *type III secretion system for therapeutic cancer vaccines J Clin Invest 2006 116 7 1946 1954 10.1172/JCI28045 16794737 PMC1481660

[B60] Widmaier DM Tullman-Ercek D Mirsky EA Hill R Govindarajan S Minshull J Voigt CA Engineering the *Salmonella *type III secretion system to export spider silk monomers Mol Syst Biol 2009 5 309 19756048 10.1038/msb.2009.62 PMC2758716

[B61] Georgiou G Segatori L Preparative expression of secreted proteins in bacteria: status report and future prospects Curr Opin Biotechnol 2005 16 5 538 545 10.1016/j.copbio.2005.07.008 16095898

[B62] Westerlund-Wikström B Tanskanen J Virkola R Hacker J Lindberg M Skurnik M Korhonen TK Functional expression of adhesive peptides as fusions to *Escherichia coli *flagellin Protein Eng 1997 10 11 1319 1326 10.1093/protein/10.11.1319 9514121

[B63] Bolivar F Rodriguez RL Greene PJ Betlach MC Heyneker HL Boyer HW Construction and characterization of new cloning vehicles. II. A multipurpose cloning system Gene 1977 2 2 95 113 10.1016/0378-1119(77)90000-2 344137

[B64] Blomfield IC McClain MS Eisenstein BI Type 1 fimbriae mutants of *Escherichia coli *K12: characterization of recognized afimbriate strains and construction of new *fim *deletion mutants Mol Microbiol 1991 5 6 1439 1445 10.1111/j.1365-2958.1991.tb00790.x 1686292

[B65] Sambrook J Russell DW Molecular cloning: a laboratory manual 2001 3 Cold Spring Harbor, NY: Cold Spring Harbor Laboratory

[B66] Westerlund B Kuusela P Risteli J Risteli L Vartio T Rauvala H Virkola R Korhonen TK The O75X adhesin of uropathogenic *Escherichia coli *is a type IV collagen-binding protein Mol Microbiol 1989 3 3 329 337 10.1111/j.1365-2958.1989.tb00178.x 2568575

[B67] Karlsson R Katsamba PS Nordin H Pol E Myszka DG Analyzing a kinetic titration series using affinity biosensors Anal Biochem 2006 349 1 136 147 10.1016/j.ab.2005.09.034 16337141

[B68] Blattner FR Plunkett G Bloch CA Perna NT Burland V Riley M Collado-Vides J Glasner JD Rode CK Mayhew GF Gregor J Davis NW Kirkpatrick HA Goeden MA Rose DJ Mau B Shao Y The complete genome sequence of *Escherichia coli *K-12 Science 1997 277 5331 1453 1474 10.1126/science.277.5331.1453 9278503

[B69] Sutcliffe JG Complete nucleotide sequence of the *Escherichia coli *plasmid pBR322 Cold Spring Harb Symp Quant Biol 1979 43 77 90 383387 10.1101/sqb.1979.043.01.013

[B70] Gasteiger E Hoogland C Gattiker A Duvaud S Wilkins MR Appel RD Bairoch A Walker JM Protein identification and analysis tools on the ExPASy Server The Proteomics Protocols Handbook 2005 Humana Press 571 607

[B71] Bendtsen JD Nielsen H von Heijne G Brunak S Improved prediction of signal peptides: SignalP 3.0 J Mol Biol 2004 340 4 783 795 10.1016/j.jmb.2004.05.028 15223320

[B72] Juncker AS Willenbrock H Von Heijne G Brunak S Nielsen H Krogh A Prediction of lipoprotein signal peptides in Gram-negative bacteria Protein Sci 2003 12 8 1652 1662 10.1110/ps.0303703 12876315 PMC2323952

[B73] Kankainen M Blannotator http://ekhidna.biocenter.helsinki.fi/poxo/blannotator

