# Supplementary material for: Adhesive polypeptides of Staphylococcus aureus identified using a novel secretion library technique in Escherichia coli
Source: BMC Microbiol. 2011 May 27;11:117. doi: 10.1186/1471-2180-11-117 (PMC3127751; doi:10.1186/1471-2180-11-117)
Supplement: Additional file 1 — "Table S1" shows the list of gene products found by DNA sequencing and bioinformatics of the Ftp-library. Examples of known adhesive surface proteins and adhesive polypeptides described in the current report are shown in boldface. The abbreviations used as clone and polypeptide names in the current report are shown in parenthesis. [file 1471-2180-11-117-S1.PDF]

Table S1. List of gene products identified in the Ftp library. Known adhesive surface proteins and adhesive polypeptides described in the current report, are shown in boldface. The abbreviations used as clone and polypeptide names in the report are shown in parenthesis.

No.	GI	Blannotator Annotation
1	88196627	1-(5-phosphoribosyl)-5-[(5- phosphoribosylamino)methylideneamino] imidazole-4-carboxamide isomerase
2	88194737	1,4-dihydroxy-2-naphthoate octaprenyltransferase
3	88195537	1-acyl-sn-glycerol-3-phosphate acyltransferase
4	88196500	1-pyrroline-5-carboxylate dehydrogenase
5	88195124	2,3,4,5-tetrahydropyridine-2-carboxylate N-succinyltransferase, putative
6	88196343	2,3-bisphosphoglycerate-dependent phosphoglycerate mutase
7	88194558	2,3-bisphosphoglycerate-independent phosphoglycerate mutase
8	88193920	2',3'-cyclic-nucleotide 2'-phosphodiesterase
**9**	**88194032**	**2-C-methyl-D-erythritol 4-phosphate cytidylyltransferase (IspD)**
10	88195955	2-isopropylmalate synthase
11	88194671	2-isopropylmalate synthase, putative
12	88195143	2-oxoglutarate dehydrogenase E1 component
13	88195142	2-oxoglutarate dehydrogenase, E2 component, dihydrolipoamide succinyltransferase
14	88194740	2-succinyl-5-enolpyruvyl-6-hydroxy-3-cyclohexene-1- carboxylate synthase
15	88195611	2-succinylbenzoate--CoA ligase
16	88196138	30S ribosomal protein S13
17	88194965	30S ribosomal protein S2
18	88195395	30S ribosomal protein S20
19	88195664	3'-5' exoribonuclease yhaM
20	88194590	3-dehydroquinate dehydratase
21	88195199	3-dehydroquinate synthase
22	88194332	3-hexulose-6-phosphate synthase
23	88194006	3-hydroxyacyl-CoA dehydrogenase
24	88195957	3-isopropylmalate dehydratase large subunit
25	88195956	3-isopropylmalate dehydrogenase
26	88195198	3-phosphoshikimate 1-carboxyvinyltransferase
27	88196510	4,4'-diaponeurosporene oxidase
28	88195076	4-hydroxybenzoyl-CoA thioesterase
29	88193957	4'-phosphopantetheinyl transferase superfamily protein
30	88194299	50S ribosomal protein L11
31	88196159	50S ribosomal protein L2
32	88194261	50S ribosomal protein L25
33	88196162	50S ribosomal protein L3
34	88196024	50S ribosomal protein L31
35	88194138	5-methyltetrahydropteroyltriglutamate--homocysteine methyltransferase
36	88195408	5'-methylthioadenosine/S-adenosylhomocysteine nucleosidase
37	88194617	5'-nucleotidase family protein
38	88195582	6,7-dimethyl-8-ribityllumazine synthase
39	88196101	6-phospho-beta-galactosidase
40	88195316	6-phosphogluconate dehydrogenase, decarboxylating
41	88195693	A/G-specific adenine glycosylase
42	88193977	ABC transporter ATP-binding protein
43	88194513	ABC transporter ATP-binding protein

No.	GI	Blannotator Annotation
44	88195641	ABC transporter ATP-binding protein
45	88196260	ABC transporter ATP-binding protein
46	88195141	ABC transporter homolog
47	88194733	ABC transporter periplasmic binding protein, putative
**48**	**88196085**	**ABC transporter periplasmic binding protein, putative (PBP)**
49	88194216	ABC transporter, ATP-binding protein
50	88194494	ABC transporter, ATP-binding protein
51	88195364	ABC transporter, ATP-binding protein
52	88195659	ABC transporter, ATP-binding protein
53	88196281	ABC transporter, ATP-binding protein
54	88196637	ABC transporter, ATP-binding protein, putative
55	88193988	ABC transporter, permease protein
56	88194011	ABC transporter, substrate-binding protein
57	88194150	Abi family protein
58	88195935	Accessory gene regulator A
59	88196606	Accessory secretory protein Asp2
60	88195520	Acetate kinase
61	88193901	Acetoin reductase
62	88195547	Acetoin utilization protein AcuC
63	88195952	Acetolactate synthase
64	88196120	Acetolactate synthase, putative
65	88194337	Acetyl-CoA acetyltransferase
66	88195334	Acetyl-CoA carboxylase, biotin carboxyl carrier protein
67	88195333	Acetyl-CoA carboxylase, biotin carboxylase
68	88195415	Acetyl-CoA carboxylase, biotin carboxylase, putative
69	88195509	Acetyl-CoA carboxylase, carboxyl transferase, beta subunit
70	88195545	Acetyl-CoA synthetase
71	88196293	Acetyltransferase, GNAT family
72	88196469	Acetyltransferase, GNAT family
73	88196461	Acetyltransferase, GNAT family protein
74	88195075	Aconitate hydratase
75	88196355	Adenosylmethionine-8-amino-7-oxononanoate aminotransferase
76	88196141	Adenylate kinase
77	88195807	Adenylosuccinate lyase
78	88195010	Aerobic glycerol-3-phosphate dehydrogenase
79	88195518	Alanine dehydrogenase
80	88195971	Alanine racemase
81	88194894	Alanine racemase domain protein
82	88196087	Alanine racemase domain protein
83	88195427	Alanyl-tRNA synthetase
84	88194378	Alcohol dehydrogenase
85	88196026	Aldehyde dehydrogenase
86	88193945	Aldehyde dehydrogenase homolog
87	88196256	Aldose 1-epimerase
88	88196581	Alkaline phosphatase
89	88194931	Alkaline shock protein
90	88194162	Alkyl hydroperoxide reductase subunit F
91	88196410	Alkylhydroperoxidase, AhpD family
92	88195418	Allophanate hydrolase subunit 1
93	88195016	Aluminum resistance protein
94	88193877	Amidohydrolase
95	88194311	Amidohydrolase
96	88196037	Amidohydrolase

No.	GI	Blannotator Annotation
97	88196339	Amino acid ABC transporter, amino acid-binding protein
98	88196337	Amino acid ABC transporter, ATP-binding protein, putative
99	88196550	Amino acid transporter
100	88195101	Aminoacyltransferase femB
101	88196174	Aminoacyltransferase femX
102	88195775	Aminopeptidase
103	88195838	Aminotransferase
104	88196506	Aminotransferase
105	88195525	Aminotransferase class V
106	88195532	Aminotransferase, class V
107	88196568	Anaerobic ribonucleoside-triphosphate reductase
108	88194463	Anion transporter
109	88196647	Anion transporter
110	88195095	Anthranilate phosphoribosyltransferase
111	88195093	Anthranilate synthase component I
112	88194107	Appr-1-p processing domain protein
113	88196069	Arginase
114	88196591	Arginine repressor
115	88194656	Argininosuccinate synthase
116	88194381	Arginyl-tRNA synthetase
117	88194906	Aspartate carbamoyltransferase
118	88195798	Aspartyl/glutamyl-tRNA(Asn/Gln) amidotransferase subunit B
119	88195800	Aspartyl/glutamyl-tRNA(Asn/Gln) amidotransferase subunit C
120	88195441	Aspartyl-tRNA synthetase
121	88196011	ATP synthase B chain
122	88196010	ATP synthase delta chain
123	161353520	ATP synthase gamma chain
124	88196009	ATP synthase subunit alpha
125	88194285	ATP:guanido phosphotransferase
126	161353524	ATPase AAA family protein
127	88195479	ATP-dependent Clp protease ATP-binding subunit clpX
128	88195804	ATP-dependent DNA helicase pcrA
129	88194495	ATP-dependent DNA helicase RecQ
130	88194662	ATP-dependent nuclease subunit A, putative
131	88195366	ATP-dependent RNA helicase
132	88195982	ATP-dependent RNA helicase
133	88196592	Aureolysin
134	88194105	Bacterial luciferase family protein
135	88194120	Bacterial luciferase family protein
136	88193857	Beta-lactamase domain protein
137	88194750	Bifunctional autolysin
138	88194761	Bifunctional protein folD
139	88194258	Bifunctional protein glmU
140	88194771	Bifunctional purine biosynthesis protein purH
141	88196238	Bile acid:sodium symporter
142	88196621	Biofilm PGA synthesis N-glycosyltransferase PgaC
143	88196623	Biofilm PIA synthesis deacetylase icaB
144	88196622	Biofilm PIA synthesis protein icaD
145	88196354	Biotin synthase
146	88195322	Branched-chain alpha-keto acid dehydrogenase E2
147	88194316	Branched-chain amino acid aminotransferase
148	88193963	Branched-chain amino acid transport system II carrier protein
149	88194086	Branched-chain amino acid transport system II carrier protein

No.	GI	Blannotator Annotation
150	88195640	BsaE protein
151	88193936	Capsular polysaccharide biosynthesis protein Cap5J
152	88193927	Capsular polysaccharide biosynthesis protein, putative
153	88193932	Capsular polysaccharide synthesis enzyme
154	88193938	Capsular polysaccharide synthesis enzyme Cap5L
155	88193940	Capsular polysaccharide synthesis enzyme Cap5N
156	88193934	Capsular polysaccharide synthesis enzyme O-acetyl transferase Cap5H
157	88194872	Carbamate kinase
158	88196587	Carbamate kinase
159	88194909	Carbamoyl-phosphate synthase large chain
160	88194562	Carboxylesterase
161	88195151	Carboxyl-terminal protease
162	88195989	Cardiolipin synthetase
163	88195057	Catalase
164	88195372	CBS domain protein
165	88194993	CDP-diacylglycerol--glycerol-3-phosphate 3-phosphatidyltransferase
166	88195985	Cell division membrane protein
167	88194886	Cell division protein
168	88194890	Cell division protein
169	88194892	Cell division protein ftsZ
170	88194895	Cell division protein sepF
171	88195388	Chaperone protein dnaJ
172	88195389	Chaperone protein dnaK
173	88196559	Choline dehydrogenase
174	88195200	Chorismate synthase
175	88195492	Chromosome replication initiation/membrane attachment protein
176	88194941	Chromosome segregation SMC protein, putative
**177**	**88194572**	**Clumping factor A, Fragment**
**178**	**88196585**	**Clumping factor B, Fragment**
179	88194204	Cobalamin synthesis protein P47K
180	88196529	Cobalamin synthesis protein/P47K family protein
181	88196133	Cobalt import ATP-binding protein cbiO 1
182	88194773	Cobalt transport protein
183	88194843	Colicin V production protein
184	88194819	ComK regulator
185	88194693	Competence CoiA family protein
186	88194994	Competence-damage inducible protein cinA
187	88196423	Conserved domain protein
188	88196123	Conserved domain protein, putative
189	88195294	Conserved hypothetical phage protein
190	88193999	conserved protein
191	88194123	conserved protein
192	88194399	conserved protein
193	88194527	conserved protein
194	88194532	conserved protein
195	88195068	conserved protein
196	88195346	conserved protein
197	88195580	conserved protein
198	88195960	conserved protein
199	88196086	conserved protein
200	88196180	conserved protein
201	88196200	conserved protein
202	88196278	conserved protein

No.	GI	Blannotator Annotation
203	88196333	conserved protein
204	88196371	conserved protein
205	88196448	conserved protein
206	88196530	conserved protein
207	88196609	conserved protein
208	88196659	conserved protein
209	88196672	conserved protein
210	88196677	conserved protein
211	88196686	conserved protein
212	88196711	conserved protein
213	88195161	Conserved virulence factor C
214	88196504	Copper chaperone copZ
215	88196503	Copper-translocating P-type ATPase
216	88196265	CorA-like Mg2+ transporter protein
217	88194603	CsbD family protein
218	88196031	CTP synthase
219	88194140	Cystathionine beta-lyase
220	88194141	Cystathionine gamma-synthase
221	88194215	Cystathionine gamma-synthase
222	88194214	Cysteine synthase
223	88194273	Cysteine synthase
224	88194292	Cysteinyl-tRNA synthetase
225	88194636	Cytosol aminopeptidase
226	88195533	D-3-phosphoglycerate dehydrogenase
227	88194932	Dak phosphatase
228	88194626	D-alanine--poly(phosphoribitol) ligase subunit 1
229	88195157	DegV family protein
230	88195473	Delta-aminolevulinic acid dehydratase
231	88193913	Deoxyribose-phosphate aldolase
232	88196042	Deoxyribose-phosphate aldolase
233	88193837	DHH subfamily 1 protein
234	88195796	Diacylglycerol kinase
235	88193897	Diaminopimelate decarboxylase
236	88195127	Diaminopimelate decarboxylase
237	88196406	Diaminopimelate epimerase-like protein
238	88195123	Dihydrodipicolinate reductase
239	88195158	Dihydrofolate reductase
240	88194795	Dihydrolipoyl dehydrogenase
241	88195325	Dihydrolipoyl dehydrogenase
242	88194275	Dihydroneopterin aldolase
243	88194907	Dihydroorotase
244	88196537	Dihydroorotate dehydrogenase
245	88194274	Dihydropteroate synthase
246	88195951	Dihydroxy-acid dehydratase
247	88195686	D-isomer specific 2-hydroxyacid dehydrogenase family protein
248	88196505	D-lactate dehydrogenase, putative
249	88193829	DNA gyrase subunit A
250	88195803	DNA ligase
251	88195006	DNA mismatch repair protein mutL
252	88195005	DNA mismatch repair protein mutS
253	88195497	DNA polymerase I
254	88194974	DNA polymerase III polC-type
255	88195511	DNA polymerase III, alpha subunit

No.	GI	Blannotator Annotation
256	88195396	DNA polymerase III, delta subunit
257	88194233	DNA polymerase III, gamma and tau subunits, putative
258	88195793	DNA polymerase IV
259	88195370	DNA primase
260	88195326	DNA repair protein RecN
261	88194958	DNA topoisomerase
262	88194986	DNA translocase ftsK
263	88195297	DNA-binding response regulator
264	88194305	DNA-directed RNA polymerase
265	161353532	DNA-directed RNA polymerase , fragment
266	88196340	Drug resistance transporter, EmrB/QacA family protein
267	88196077	Drug transporter, putative
268	88195174	Dynamin family protein
**269**	**88195217**	**Elastin-binding protein ebpS**
270	88194309	Elongation factor G
271	88194967	Elongation factor Ts
272	88195186	Endonuclease III
**273**	**88194559**	**Enolase**
274	88196375	Epimerase/dehydratase, putative
275	88196477	Esterase
276	88195613	Excalibur domain protein
277	88194540	Excinuclease ABC subunit B, putative
278	88195329	Exodeoxyribonuclease 7 small subunit
279	88194181	Exotoxin
280	88194190	Exotoxin
281	88194194	Exotoxin
**282**	**88194575**	**Extracellular matrix protein-binding protein emp**
**283**	**88195168**	**Extracellular matrix-binding protein ebhA (Ebh)**
284	88193858	FAD-dependent pyridine nucleotide-disulphide oxidoreductase
285	88196532	FAD-dependent pyridine nucleotide-disulphide oxidoreductase
286	88195654	Ferrochelatase
**287**	**88196438**	**Fibronectin binding protein A (FnBPA)**
**288**	**88196437**	**Fibronectin binding protein A , fragment**
**289**	**88194914**	**Fibronectin-binding protein**
290	88196065	FmtB
291	88195570	FmtB-like protein
292	88195663	Foldase protein prsA
293	88195467	Folylpolyglutamate synthase
294	88193997	Formate acetyltransferase
295	88196225	Formate dehydrogenase homolog
296	88196327	Formate/nitrite transporter, putative
297	88195544	Formate--tetrahydrofolate ligase
298	88196252	Formimidoylglutamase
299	88196553	Fructose-bisphosphate aldolase
300	88195555	FtsK/SpoIIIE family protein
301	88195674	Fumarate hydratase class II
302	88196106	Galactose-6-phosphate isomerase subunit lacB
303	88193981	Gamma-glutamyltranspeptidase
304	88196350	Gamma-hemolysin component B
305	88195328	Geranyltranstransferase
306	88195354	Glucokinase
307	88196443	Gluconate operon transcriptional repressor, putative
308	88195678	Glucosamine-6-phosphate isomerase, putative

No.	GI	Blannotator Annotation
309	88196060	Glucosamine--fructose-6-phosphate aminotransferase [isomerizing]
310	88194657	Glucose-6-phosphate isomerase
311	88194852	Glutamate racemase
312	88194226	Glutamate synthase, large subunit, putative
313	88195472	Glutamate-1-semialdehyde 2,1-aminomutase
314	88195559	Glutamyl aminopeptidase
315	88195799	Glutamyl-tRNA(Gln) amidotransferase subunit A
316	88196574	Glutathione peroxidase
317	88194555	Glyceraldehyde-3-phosphate dehydrogenase
318	88195494	Glyceraldehyde-3-phosphate dehydrogenase
319	88196363	Glycerate kinase
320	88195009	Glycerol kinase
321	88194118	Glycerol-3-phosphate transporter
322	88194000	Glycerophosphoryl diester phosphodiesterase
323	88195074	Glycine betaine transporter
324	88196381	Glycine betaine/carnitine/choline ABC transporter, permease protein
325	88196382	Glycine betaine/L-proline ABC transporter, ATP-binding protein
326	88194731	Glycosyl transferase, group 1
327	88195192	Glycosyl transferase, group 1
328	88196603	Glycosyl transferase, group 1 family protein
329	88196511	Glycosyl-4,4'-diaponeurosporenoate acyltransferase
330	88194119	Glyoxalase family protein
331	88196462	Glyoxalase family protein
332	88195060	GMP reductase
333	88196602	GtfB
334	88195445	GTP pyrophosphokinase
335	161353527	GTPase
336	88195394	GTP-binding protein lepA
337	88195406	GTP-binding protein, putative
338	88196117	HAD-superfamily hydrolase subfamily IIB
339	88195535	Haloacid dehalogenase-like hydrolase
340	88194376	HD domain protein
341	88195990	HD domain protein
342	88196310	Heat shock protein
343	88196426	Helicase, putative
344	88195428	Helicase, putative, RecD/TraA family
345	88196284	Heme sensor protein hssS
346	88193943	Heme-degrading monooxygenase isdG
347	88195203	Heptaprenyl diphosphate synthase component II
348	161353531	Hexulose-6-phosphate isomerase
349	88195569	HI0933 family protein
350	88195125	Hippurate hydrolase
351	88196630	Histidinol-phosphate aminotransferase, putative
352	88195442	Histidyl-tRNA synthetase
353	88195660	HIT family protein
354	88195183	Holliday junction resolvase recU
355	88195050	Homoserine dehydrogenase
356	88195052	Homoserine kinase
357	88193882	HTH-type transcriptional regulator norG
358	88195576	HTH-type transcriptional regulator rot
359	88196309	HTH-type transcriptional regulator sarZ
360	88196115	Hyaluronate lyase
361	88194385	Hydrolase

No.	GI	Blannotator Annotation
362	88196528	Hydrolase
363	88195930	Hydrolase, carbon-nitrogen family
364	88196491	Hydroxymethylglutaryl-CoA synthase
365	88194550	Hypothetical cytosolic protein
366	88194198	Hypothetical lipoprotein
367	88194199	Hypothetical lipoprotein
368	88193883	Hypothetical membrane protein
369	88194060	Hypothetical membrane protein
370	88194075	Hypothetical membrane protein
371	88194679	Hypothetical membrane protein
372	88194705	Hypothetical membrane protein
373	88194802	Hypothetical membrane protein
374	88194816	Hypothetical membrane protein
375	88195109	Hypothetical membrane protein
376	88195662	Hypothetical membrane protein
377	88196269	Hypothetical membrane protein
378	88196296	Hypothetical membrane protein
379	88196351	Hypothetical membrane protein
380	88196662	Hypothetical membrane protein
381	88194552	Hypothetical sugar nucleotide epimerase
382	88194270	Hypoxanthine phosphoribosyltransferase
383	88196626	Imidazole glycerol phosphate synthase subunit hisF
384	88196628	Imidazole glycerol phosphate synthase subunit hisH
385	88196248	Imidazolonepropionase
386	88196593	Immunodominant staphylococcal antigen B
**387**	**88193885**	**Immunoglobulin G binding protein A**
**388**	**88196346**	**Immunoglobulin-binding protein sbi**
389	88194093	Indigoidine synthase A family protein
390	88193965	Indole-3-pyruvate decarboxylase
391	88194806	Inositol monophosphatase family protein
392	88193881	Integral membrane domain protein
393	88194151	Integrase-like protein
394	88195701	Intracellular protease, PfpI family
395	88195918	Iron compound ABC transporter, iron compound-binding protein
396	88196199	Iron compound ABC transporter, iron compound-binding protein
397	88193996	Iron compound ABC transporter, iron compound-binding protein, putative
398	88194834	Iron compound ABC transporter, permease protein
399	88194405	Iron dependent repressor
**400**	**88194828**	**Iron-regulated surface determinant protein B**
401	88194830	Iron-regulated surface determinant protein C
**402**	**88195542**	**Iron-regulated surface determinant protein H**
403	88195136	IS1272-like transposase, degenerate
404	88195819	Isochorismatase family protein
405	88195501	Isocitrate dehydrogenase [NADP]
406	88194899	Isoleucyl-tRNA synthetase
407	88196088	IucA/IucC family protein
408	88196090	IucA/IucC family protein
409	88195977	K+-transporting ATPase, B subunit
410	88196641	Lactonase drp35
411	88195644	Lantibiotic epidermin biosynthesis protein EpiC
412	88195214	L-asparaginase
413	88195572	Leucyl-tRNA synthetase
414	88194101	Lipase

No.	GI	Blannotator Annotation
415	88196625	Lipase
416	88195223	Lipoprotein
417	88196419	Lipoprotein
418	88196424	Lipoprotein
419	88196425	Lipoprotein
420	88195225	Lipoprotein, putative
421	88195226	Lipoprotein, putative
422	88194218	Lipoprotein, YaeC family
423	88196549	L-lactate dehydrogenase
424	88196472	L-serine dehydratase, iron-sulfur-dependent, alpha subunit
425	88195433	Luciferase family protein
426	88196250	LysR family regulatory protein
427	88194278	Lysyl-tRNA synthetase
428	88196053	Lytic regulatory protein
429	88194702	Magnesium transporter
430	88196449	Major facilitator superfamily MFS_1
431	88196287	Malate:quinone oxidoreductase
432	88194936	Malonyl CoA-acyl carrier protein transacylase
433	88196064	Mannitol-1-phosphate 5-dehydrogenase
434	88196063	Mannitol-specific phosphotransferase enzyme IIA component
435	88196048	Mannose-6-phosphate isomerase
436	88194725	Membrane protein
437	88195993	Membrane protein oxaA
438	88195942	Membrane protein, YeeE/YedE family
439	88196276	Membrane-associated protein tcaA
440	88195204	Menaquinone biosynthesis methyltransferase ubiE
441	88196330	Metal-binding protein
442	88195515	Metal-dependent hydrolase
443	88194985	Metallo-beta-lactamase family protein
444	88194956	Methicillin resistance protein
445	88195100	Methicillin resistance protein FemA
446	88194922	Methionyl-tRNA formyltransferase
447	88194248	Methionyl-tRNA synthetase, putative
448	88194139	Methylenetetrahydrofolate reductase
449	88194959	Methylenetetrahydrofolate--tRNA-(uracil-5-)- methyltransferase trmFO
450	88194303	Methyltransferase small
451	88195840	MHC class II analog protein
452	88196021	Modification methylase, HemK family, putative
453	88196188	Molybdopterin biosynthesis protein moeA, putative
454	88196191	Molybdopterin biosynthesis protein MoeB
455	88195789	Mur ligase family protein
456	88194845	MutS2 family protein
457	88193952	MW0149 protein
458	88195205	MW1361 protein
459	88193879	Na/Pi-cotransporter family protein
460	88194397	Na+/H+ antiporter, MnhD component, putative
461	88193961	N-acetyl-gamma-glutamyl-phosphate reductase
462	88193969	N-acetylmuramic acid 6-phosphate etherase
463	88196599	N-acetylmuramoyl-L-alanine amidase domain protein
464	88194219	N-acetylmuramoyl-L-alanine amidase sle1
465	88196050	NAD-dependent epimerase/dehydratase
466	88194650	NADH-dependent flavin oxidoreductase
467	88194742	Naphthoate synthase

No.	GI	Blannotator Annotation
468	88195812	NH(3)-dependent NAD(+) synthetase
469	161353521	Nicotinate phosphoribosyltransferase
470	88196320	Nitrate reductase beta subunit
471	88196321	Nitrate reductase, alpha subunit (NarG)
472	88194179	NmrA family protein
473	88193956	Non-ribosomal peptide synthetase
474	88196317	NreA protein
475	88195071	Nuclease sbcCD subunit C
476	88195070	Nuclease sbcCD subunit D
477	88194282	Nucleoside transporter NupC
478	88194415	Nucleoside transporter NupC
479	88194668	O-acetyltransferase oatA
480	88195312	Oligo-1,6-glucosidase
481	88194682	Oligopeptide ABC transporter, ATP-binding protein
482	88194687	Oligopeptide ABC transporter, ATP-binding protein
483	88196399	Oligopeptide ABC transporter, ATP-binding protein
484	88194681	Oligopeptide ABC transporter, permease protein
485	88196403	Oligopeptide transporter putative substrate binding domain
486	88194237	Orn/Lys/Arg decarboxylase
487	88193962	Ornithine aminotransferase, putative
488	88196589	Ornithine carbamoyltransferase
489	88194911	Orotate phosphoribosyltransferase
490	88195531	OsmC/Ohr family protein
491	88195610	O-succinylbenzoic acid synthetase
492	88193989	Oxidoreductase domain protein
493	88193990	Oxidoreductase domain protein
494	88194990	Oxidoreductase, short-chain dehydrogenase/reductase family
495	88196099	Oxidoreductase, zinc-binding dehydrogenase family
496	88196292	Oxidoreductase, zinc-binding dehydrogenase family protein
497	88196316	Oxygen sensor histidine kinase nreB
498	88196545	Pantothenate synthetase
499	88194210	PAP2 family protein
500	88195818	Parallel beta-helix repeat
501	88195360	Penicillin-binding protein
502	88195184	Penicillin-binding protein 2
503	88194413	Penicillin-binding protein 4
504	88195826	Peptidase C45 acyl-coenzyme A:6-aminopenicillanic acid acyl-transferase
505	88194521	Peptidase T
506	88196391	Peptidase, M42 family
507	88196022	Peptide chain release factor 1
508	88194533	Peptide chain release factor 2
509	88194713	Peptide chain release factor 3
510	88195087	Peptide methionine sulfoxide reductase
511	88195155	Peptide methionine sulfoxide reductase msrB
512	88194092	PfkB family carbohydrate kinase
513	88196598	Phage infection protein, putative
514	88194839	Phenylalanyl-tRNA synthetase alpha chain
515	88195380	PhoH family protein
516	88195112	Phosphate ABC transporter, ATP-binding protein
517	88195113	Phosphate ABC transporter, permease protein, putative
518	88195116	Phosphate binding protein
519	88194435	Phosphate transporter
520	88195550	Phospho-2-dehydro-3-deoxyheptonate aldolase

No.	GI	Blannotator Annotation
521	88195605	Phosphoenolpyruvate carboxykinase
522	88194782	Phosphoenolpyruvate-protein phosphotransferase
523	88196428	Phosphoglucomutase
524	88196066	Phosphoglucosamine mutase
525	88194556	Phosphoglycerate kinase
526	88195996	Phosphomethylpyrimidine kinase
527	88194353	Phosphomevalonate kinase
528	88194888	Phospho-N-acetylmuramoyl-pentapeptide-transferase
529	88194824	Phosphopantetheine adenylyltransferase
530	88194917	Phosphopantothenoylcysteine decarboxylase/phosphopantothenate--cysteine ligase
531	88193914	Phosphopentomutase
**532**	**88194763**	**Phosphoribosylaminoimidazole carboxylase, ATPase subunit (PurK)**
533	88194764	Phosphoribosylaminoimidazole-succinocarboxamide synthase
534	88194769	Phosphoribosylformylglycinamidine cyclo-ligase
535	88194766	Phosphoribosylformylglycinamidine synthase I
536	88194767	Phosphoribosylformylglycinamidine synthase II
537	88194770	Phosphoribosylglycinamide formyltransferase
538	88194844	PHP domain protein
539	88194289	PilT protein domain protein
540	88194984	Polyribonucleotide nucleotidyltransferase
541	88195475	Porphobilinogen deaminase
542	88193846	Possible 5'-nucleotidase
543	88195470	Possible ammonia monooxygenase
544	88195946	Possible DNA mismatch repair protein MutS
545	88196078	Possible hemolysin III
546	88195102	Possible hydrolase
547	88195295	Possible lipoprotein
548	88193867	Possible transcriptional regulator
549	88193860	Possible tRNA-dihydrouridine synthase
550	88195978	Potassium-transporting ATPase A chain
551	88193958	Predicted periplasmic/secreted protein
552	88195091	Prephenate dehydrogenase
553	88194250	Primase-related protein
554	88194613	Probable 2-nitropropane dioxygenase
555	88195189	Probable ATP-dependent helicase dinG homolog
556	88195548	Probable catabolite control protein A
557	88195443	Probable cell wall amidase lytH
558	88194195	Probable exported protein
559	88196502	Probable exported protein
560	88195478	Probable GTP-binding protein engB
561	88195086	Probable lysylphosphatidylglycerol synthetase
562	88195820	Probable manganese-dependent inorganic pyrophosphatase
563	88194406	Probable membrane protein
564	88194523	Probable membrane protein
565	88194526	Probable membrane protein
566	88195085	Probable membrane protein
567	88195383	Probable membrane protein
568	88194103	Probable NADH-dependent flavin oxidoreductase
569	88196480	Probable PTS system
570	88194758	Probable quinol oxidase subunit 2
571	88195538	Probable serine protease
572	88195642	Probable serine protease
573	88194265	Probable tetrapyrrole methylase

No.	GI	Blannotator Annotation
574	88195524	Probable thiamine biosynthesis protein thiI
575	88193876	Probable transcriptional regulator
576	88194459	Probable transcriptional regulator MarR family
577	88195540	Probable transglycosylase
578	88196515	Probable transglycosylase isaA
579	88195999	Probable transglycosylase sceD
580	88195581	Proline dehydrogenase
581	88194973	Prolyl-tRNA synthetase
582	88194009	Propionate CoA-transferase, putative
583	88195598	Protein crcB homolog
584	88194064	Protein esaA
585	88194066	Protein esaB
586	88194068	Protein essC
587	88196195	Protein fdhD homolog
588	88195390	Protein grpE
589	88194926	Protein kinase
590	88194506	Protein nrdI
591	88194531	Protein translocase subunit secA
592	88196604	Protein translocase subunit secA
593	88194815	Protoheme IX farnesyltransferase
594	88196307	Proton/sodium-glutamate symport protein
595	88194903	Pseudouridine synthase
596	88195298	Pseudouridine synthase
597	88195567	Pseudouridine synthase
598	88196596	Pts system fructose-specific eiibc component
599	88194473	PTS system fructose-specific IIABC component
600	88193967	PTS system glucose-specific EIICBA component
601	88196301	PTS system sucrose-specific IIBC component, putative
602	88194042	PTS system, IIA component
603	88194113	PTS system, IIA component
604	88194018	Pts system, iibc component
605	88196102	PTS system, lactose-specific IIBC component
606	88196061	PTS system, mannitol-specific component, putative
607	88195536	PTS system, N-acetylglucosamine-specific IIBC component
608	88193970	PTS system, sucrose-specific, IIBC component
609	88194228	PTS system, trehalose-specific IIBC component
610	88196262	Putative 3-methyladenine DNA glycosylase
611	88196353	Putative 8-amino-7-oxononanoate synthase
612	88196359	Putative ABC transporter
613	88195150	Putative acetyltransferase
614	88194007	Putative acyl-CoA dehydrogenase
615	88194008	Putative acyl-CoA synthetase
616	88194029	Putative alcohol dehydrogenase
617	88194033	Putative alcohol dehydrogenase
618	88193926	Putative aldehyde-alcohol dehydrogenase
619	88196386	Putative amino acid permease
620	88194487	Putative anthranilate synthase component II
621	88196014	Putative ATP synthase protein I
622	88196560	Putative betaine aldehyde dehydrogenase
623	88194812	Putative cell division protein
624	88194271	Putative cell division protein FtsH
625	88195350	Putative competence protein ComGB
626	88195432	Putative cysteine desulfurase

No.	GI	Blannotator Annotation
627	88194321	Putative deaminase
628	88196218	Putative dehydrogenase
629	88194501	Putative diacylglycerol kinase protein
630	88194421	Putative dihydroxyacetone kinase
631	88195666	Putative DNA repair exonuclease
632	88195019	Putative DNA-binding protein
633	88195592	Putative DNA-binding protein
634	88196178	Putative drug transporter
635	88194255	Putative endoribonuclease L-PSP
636	88194426	Putative esterase
637	88193919	Putative exported protein
638	88194059	Putative exported protein
639	88194196	Putative exported protein
640	88194455	Putative exported protein
641	88195047	Putative exported protein
642	88195177	Putative exported protein
643	88195566	Putative exported protein
644	88195603	Putative exported protein
645	88195661	Putative exported protein
646	88195827	Putative exported protein
647	88196306	Putative exported protein
648	88196308	Putative exported protein
649	88194014	Putative flavohemoprotein
650	88195528	Putative GAF sensor protein
651	88195805	Putative glycerol-1-phosphate prenyltransferase
652	88194035	Putative glycosyl transferase
653	88194326	Putative glycosyl transferase
654	88194327	Putative glycosyl transferase
655	88194411	Putative glycosyltransferase tagX
656	88193872	Putative lipoprotein
657	88193873	Putative lipoprotein
658	88193874	Putative lipoprotein
659	88193950	Putative lipoprotein
660	88194083	Putative lipoprotein
661	88194155	Putative lipoprotein
662	88194160	Putative lipoprotein
663	88194791	Putative lipoprotein
664	88195337	Putative lipoprotein
665	88195612	Putative lipoprotein
666	88195615	Putative lipoprotein
667	88195802	Putative lipoprotein
668	88196501	Putative maltose O-acetyltransferase
669	88194010	Putative membrane protein
670	88194058	Putative membrane protein
671	88194127	Putative membrane protein
672	88194157	Putative membrane protein
673	88194358	Putative membrane protein
674	88194362	Putative membrane protein
675	88194364	Putative membrane protein
676	88194388	Putative membrane protein
677	88194425	Putative membrane protein
678	88194863	Putative membrane protein
679	88194950	Putative membrane protein

No.	GI	Blannotator Annotation
680	88195045	Putative membrane protein
681	88195175	Putative membrane protein
682	88195320	Putative membrane protein
683	88195413	Putative membrane protein
684	88195597	Putative membrane protein
685	88195614	Putative membrane protein
686	88195665	Putative membrane protein
687	88195669	Putative membrane protein
688	88195676	Putative membrane protein
689	88195831	Putative membrane protein
690	88196170	Putative membrane protein
691	88196364	Putative membrane protein
692	88196413	Putative membrane protein
693	88196576	Putative membrane protein
694	88196684	Putative membrane protein
695	88194779	Putative methyltransferase
696	88196223	Putative N-acetylmuramoyl-L-alanine amidase
697	88196400	Putative oligopeptide ABC transporter
698	88194684	Putative oligopeptide ABC transporter, oligopeptide-binding protein
699	88195105	Putative oligopeptide transport ATP-binding protein oppD2
700	88195104	Putative oligopeptide transport ATP-binding protein oppF2
701	88193891	Putative ornithine cyclodeaminase
702	88194488	Putative para-aminobenzoate synthase component I
703	88193918	Putative phosphonate ABC transporter, phosphonate-binding protein
704	88195371	Putative phosphotransferase yqfL
705	88194434	Putative pit accessory protein
706	88194553	putative protein without homology
707	88194583	putative protein without homology
708	88194728	putative protein without homology
709	88195410	putative protein without homology
710	88195543	putative protein without homology
711	88195928	putative protein without homology
712	88196295	putative protein without homology
713	88196512	putative protein without homology
714	88194632	Putative pyridine nucleotide-disulphide oxidoreductase
715	88195575	Putative rRNA methylase
**716**	**88196526**	**Putative short chain oxidoreductase (SCOR)**
717	88193892	Putative siderophore biosynthesis protein
718	88193894	Putative siderophore biosynthesis protein
719	88196664	Putative stage 0 sporulation protein J
720	88194850	Putative succinate dehydrogenase flavoprotein subunit
721	88193904	Putative sugar transferase
722	88194031	Putative teichoic acid biosynthesis protein
723	88194976	Putative transcription factor NusA
724	88196661	Putative transcriptional regulator
725	88196388	Putative transport protein
726	88196497	Putative transport protein
727	88193851	Putative uncharacterized protein (ORF CN004)
728	88194831	Putative uncharacterized protein isdD
729	88194343	Putative uncharacterized protein MW0537
730	88195054	Putative uncharacterized protein MW1219
731	88195227	Putative uncharacterized protein MW1377
732	88195586	Putative uncharacterized protein MW1712

No.	GI	Blannotator Annotation
733	88195616	Putative uncharacterized protein MW1740
734	88195620	Putative uncharacterized protein MW1743
735	88195649	Putative uncharacterized protein MW1769
736	88195650	Putative uncharacterized protein MW1770
737	88196036	Putative uncharacterized protein MW2056
738	88196046	Putative uncharacterized protein MW2065
739	88196239	Putative uncharacterized protein MW2243
740	88196303	Putative uncharacterized protein MW2300
741	88196405	Putative uncharacterized protein MW2393
742	88196535	Putative uncharacterized protein MW2507
743	88195794	Putative uncharacterized protein probable membrane protein
744	88194566	Putative uncharacterized protein SA0738
745	88194580	Putative uncharacterized protein SA0749
746	88194639	Putative uncharacterized protein SA0806
747	88195201	Putative uncharacterized protein SA1300
748	88195381	Putative uncharacterized protein SA1401
749	88195482	Putative uncharacterized protein SA1500
750	88195617	Putative uncharacterized protein SA1621
751	88196035	Putative uncharacterized protein SA1933
752	88196415	Putative uncharacterized protein SA2267
753	88196520	Putative uncharacterized protein SA2359
754	88196646	Putative uncharacterized protein SA2485
755	88194156	Putative uncharacterized protein SAS010
756	88194207	Putative uncharacterized protein ybcD
757	88194012	Putative uncharacterized protein yeaO
758	88195941	Putative uncharacterized protein yeeD
759	88194171	Putative xanthine permease
760	88194355	Pyridine nucleotide-disulfide oxidoreductase
761	88194635	Pyridine nucleotide-disulphide oxidoreductase
762	88194280	Pyridoxine biosynthesis protein
763	88196643	Pyrrolidone-carboxylate peptidase
764	88195308	Pyrroline-5-carboxylate reductase
765	88194813	Pyruvate carboxylase
766	88195323	Pyruvate dehydrogenase E1 component beta subunit
767	88194999	Pyruvate ferredoxin oxidoreductase, alpha subunit
768	88195000	Pyruvate ferredoxin oxidoreductase, beta subunit
769	88193998	Pyruvate formate-lyase activating enzyme
770	88195506	Pyruvate kinase
771	88196481	Pyruvate oxidase
772	88193878	Quinolone resistance protein norB
773	88196378	Quinolone resistance protein norB
774	88195943	Redox-sensing transcriptional repressor rex
775	88195699	Regulatory protein recX
776	88193841	Response regulator
777	88194038	Response regulator
778	88195044	Response regulator
779	88195780	Response regulator
780	88196315	Response regulator
781	88195357	Rhomboid family protein
782	88194982	Riboflavin biosynthesis protein RibF
783	88194046	Ribokinase
784	88194940	Ribonuclease 3
785	88194563	Ribonuclease R

No.	GI	Blannotator Annotation
786	88195309	Ribonuclease Z
787	88194507	Ribonucleoside-diphosphate reductase
788	88196254	Ribose-5-phosphate isomerase A
789	88194259	Ribose-phosphate pyrophosphokinase
790	88195387	Ribosomal protein L11 methyltransferase
791	88195210	Ribosomal protein S1
792	88194124	Ribosomal-protein-serine acetyltransferase, putative
793	88195386	RNA methyltransferase, RsmE family
794	88195369	RNA polymerase sigma factor
795	88194097	ROK family protein
796	88193869	SA0089 protein
797	88193948	SA0165 protein
798	88195552	SA1559 protein
799	88195604	S-adenosylmethionine synthetase
800	88194885	S-adenosyl-methyltransferase MraW
801	88196056	SAP domain protein
802	88194436	Secretory antigen SsaA-like protein
803	88195299	Segregation and condensation protein B
804	88195043	Sensor histidine kinase
805	88195672	Sensor histidine kinase
806	88194431	Sensor histidine kinase graS
807	88193842	Sensor protein
808	88195296	Sensor protein
809	88195980	Sensor protein
810	88194037	Sensor protein lytS
811	88195781	Sensor protein vraS
812	88193975	SepS16B protein
813	88195527	Septation ring formation regulator ezrA
**814**	**88194325**	**Ser-Asp rich fibrinogen-binding, bone sialoprotein-binding protein**
**815**	**88194324**	**Ser-Asp rich fibrinogen-binding, bone sialoprotein-binding protein, fragment**
816	88194291	Serine O-acetyltransferase
817	88194715	Serine protease htrA-like
818	88194925	Serine/threonine phosphatase
819	88195965	Serine-protein kinase rsbW
820	88193832	Seryl-tRNA synthetase
821	88196246	Short-chain dehydrogenase/reductase SDR
822	88194944	Signal recognition particle protein
823	88194942	Signal recognition particle-docking protein FtsY
824	88194602	Similar to ABC transporter substrate-binding protein
825	88194898	Similar to cell-division initiation protein
826	88196305	Similar to general stress protein 26
827	88195178	Similar to methyltransferase
828	88196271	Similar to multidrug resistance protein A
829	88195698	Similar to transcriptional regulator
830	88195447	Single-stranded-DNA-specific exonuclease RecJ
831	88194108	SIR2 family protein
832	88196571	Siroheme synthase
833	88194401	Sodium/hydrogen exchanger family protein
834	88196370	Sodium/hydrogen exchanger family protein
835	88194703	Sodium/hydrogen exchanger family protein/TrkA domain protein
836	88195801	Sodium/proline symporter
837	88194095	Sodium:solute symporter family protein
838	88196468	Sortase

No.	GI	Blannotator Annotation
839	88194835	Sortase B
840	88194801	Spermidine/putrescine ABC transporter, spermidine/putrescine-binding protein, putative
841	88195808	Staphopain A
842	88194744	Staphopain B
**843**	**88194002**	**Staphylocoagulase (Coa)**
**844**	**88194574**	**Staphylocoagulase, putative**
845	88194062	Staphyloxanthin biosynthesis protein
846	88194851	Succinate dehydrogenase iron-sulfur protein
847	88194953	Succinyl-CoA ligase [ADP-forming] subunit beta
848	88195939	Sucrose operon repressor
849	88194446	Sugar efflux transporter, putative
850	88196572	Sulfite reductase flavoprotein
**851**	**88196433**	**Surface protein G**
**852**	**88193909**	**Surface protein SasD, fragment**
853	88196105	Tagatose-6-phosphate kinase
854	88194408	Teichoic acids export ATP-binding protein tagH
855	88195922	Terminase small subunit
856	88196519	TetR family regulatory protein
857	88194246	Tetrapyrrole methylase family protein
858	88195046	Thermonuclease
859	88195513	Thioesterase family protein
860	88195522	Thiol peroxidase
861	88195558	Thioredoxin, putative
862	88195038	Threonine aldolase
863	88195171	Threonine dehydratase catabolic
864	88195051	Threonine synthase
865	88194238	Thymidylate kinase
866	88195080	Topoisomerase IV subunit A
867	88195079	Topoisomerase IV subunit B
868	88194598	TOPRIM domain protein
869	88195429	TPR domain protein
870	88194225	Transcription activator of glutamate synthase operon
871	88194298	Transcription antitermination protein nusG
872	88194753	Transcription antiterminator, LytR family
873	88196025	Transcription termination factor Rho
874	88196062	Transcriptional antiterminator
875	88194021	Transcriptional antiterminator BglG family protein
876	88194554	Transcriptional regulator
877	88196214	Transcriptional regulator
878	88196241	Transcriptional regulator
879	88194283	Transcriptional regulator ctsR
880	88196325	Transcriptional regulator NirR
881	88194438	Transcriptional regulator, AraC family
882	88196304	Transcriptional regulator, AraC family
883	88196051	Transcriptional regulator, ArsR family
884	88194041	Transcriptional regulator, GntR family protein
885	88194049	Transcriptional regulator, LacI family
886	88196484	Transcriptional regulator, LysR family
887	88196583	Transcriptional regulator, MarR family
888	88196444	Transcriptional regulator, MerR family
889	88196272	Transcriptional regulator, TetR family
890	88196299	Transcriptional regulator, TetR family
891	88194263	Transcription-repair coupling factor

No.	GI	Blannotator Annotation
892	88194979	Translation initiation factor IF-2
893	88196173	Transporter, AcrB/AcrD/AcrF family
894	88196584	Tributyrin esterase
895	88195480	Trigger factor
896	88194557	Triosephosphate isomerase
897	88195012	tRNA delta(2)-isopentenylpyrophosphate transferase
898	88196667	tRNA modification GTPase mnmE
899	88196131	tRNA pseudouridine synthase A
900	88194981	tRNA pseudouridine synthase B
901	88194269	tRNA(Ile)-lysidine synthase
902	88195002	tRNA-i(6)A37 thiotransferase enzyme MiaB
903	88193973	Type I restriction enzyme
904	88195628	Type I restriction-modification system, M subunit
905	88195539	Tyrosyl-tRNA synthetase
906	88193941	UDP-N-acetyl-D-mannosamine dehydrogenase
907	88196003	UDP-N-acetylglucosamine 1-carboxyvinyltransferase
908	88196028	UDP-N-acetylglucosamine 1-carboxyvinyltransferase
909	88193942	UDP-N-acetylglucosamine 2-epimerase
910	88196015	UDP-N-acetylglucosamine 2-epimerase
911	88195554	UDP-N-acetylmuramate--L-alanine ligase
912	88194889	UDP-N-acetylmuramoylalanine--D-glutamate ligase
913	88195356	Ugl protein, fragment
914	88194631	Uncharacterised conserved protein UCP010603
915	88196285	Uncharacterized HTH-type transcriptional regulator SAOUHSC_02645
916	88195914	Uncharacterized leukocidin-like protein 1
917	88195915	Uncharacterized leukocidin-like protein 2
**918**	**88195519**	**Universal stress protein family protein (Usp)**
919	161353530	UPF0042 protein
920	88194893	UPF0124 protein SACOL1200
921	88195367	UPF0135 protein SACOL1616
922	88195067	UPF0154 protein SAB1201
923	88193848	UPF0247 protein
924	88195426	UPF0297 protein LSEI_0786
925	88195668	UPF0342 protein SAR1936
926	88195557	UPF0354 protein SAHV_1729
927	88195382	UPF0365 protein MW1525
928	88194125	UPF0409 lipoprotein
929	88195553	UPF0478 protein SA1560
930	88194805	UPF0637 protein SAS1041
931	88194905	Uracil permease, putative
932	88196016	Uracil phosphoribosyltransferase
933	88194342	Uracil-DNA glycosylase
934	88196205	Urease subunit alpha
935	88194968	Uridylate kinase
936	88196249	Urocanate hydratase
937	88196322	Uroporphyrin-III C-methyltransferase, putative
938	161353528	UvrABC system protein C
939	88194284	UvrB/UvrC protein
940	88195468	Valyl-tRNA synthetase
941	88194070	Virulence factor esxB
942	88195138	von Willebrand factor, type A
943	88195336	Xaa-Pro dipeptidase
944	88195516	Xaa-Pro dipeptidase homolog

No.	GI	Blannotator Annotation
945	88193991	Xylose isomerase domain protein TIM barrel
946	88195773	YfkB-like domain protein
947	88195160	YphP
948	88195353	YqgV
949	88195332	YqhY
950	88196614	YwrF

GI, GenInfo identifier used by NCBI.

No., gene products listed are assigned by consecutive digits
